# Research Bias in Long‐Term Monitoring of Antarctic Nearshore Marine and Terrestrial Biota

**DOI:** 10.1111/gcb.70392

**Published:** 2025-08-18

**Authors:** Shae L. Jones, Diana H. King, Vonda J. Cummings, Sharon A. Robinson, Melinda J. Waterman

**Affiliations:** ^1^ Securing Antarctica's Environmental Future (SAEF) University of Wollongong Wollongong New South Wales Australia; ^2^ Environmental Futures University of Wollongong Wollongong New South Wales Australia; ^3^ New Zealand Institute for Earth Science Wellington New Zealand

**Keywords:** Antarctica, biological monitoring, birds, invertebrates, mammals, nearshore marine, terrestrial, vegetation

## Abstract

Long‐term observations are essential for ecological research, providing insights into species and ecosystem variability, processes, and responses to change. In a time of rapid global change, ecosystem modification, and emerging threats, such long‐term monitoring (LTM) is increasingly important. Antarctica is experiencing an unprecedented change that is potentially challenging for its uniquely adapted flora and fauna. This review synthesizes LTM studies of Antarctic nearshore and terrestrial biota, examining monitored species, sites, biological parameters, and environmental factors. LTM of Antarctic biota was limited (< 140 studies) and strongly biased toward charismatic megafauna (> 60% focused on penguins and marine mammals). More than half of the studies spanned > 10 years, ~80% exceeded 5 years, and ~60% included environmental data to inform biological trends. Inconsistencies in methodologies were noted, which limit the capacity for cross‐study comparisons. Changes in local and regional species' abundances, distributions, and/or functions were reported for many of the biota groups examined. LTM efforts were concentrated along the West Antarctic Peninsula, with notable gaps across East Antarctica, reflecting the varied accessibility across the continent. Based on the limitations and gaps identified in this review, we recommend LTM of Antarctic nearshore and terrestrial ecosystems should expand to include understudied key ecosystems and locations, use harmonized protocols to ensure data are comparable, and integrate environmental monitoring at biologically relevant scales. Establishing sentinel sites and facilitating international collaboration and data sharing would be a powerful approach to circum‐Antarctic monitoring. LTM is essential not only for documenting and predicting ecological responses in Antarctica but also for informing global understanding of ecosystem resilience under climate change—providing critical data for conservation, management, and policy in a rapidly transforming world.

## Graphical abstract

Antarctic wildlife is under pressure from environmental changes and human activity, but how well is Antarctica's coastal biota being monitored? While over half of the long‐term monitoring studies spanned more than 10 years and/or included environmental data, they were limited in number, mostly focused on penguins and marine mammals, were unevenly spread across Antarctica, and used different methods, making it difficult to compare results between them. To better understand the responses of and how to protect these fragile ecosystems, we need more consistent, representative, and widespread monitoring across Antarctica.

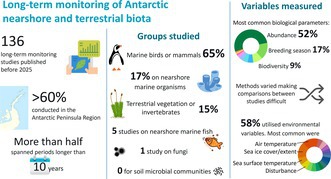

## 1 Introduction

**FIGURE 1:**
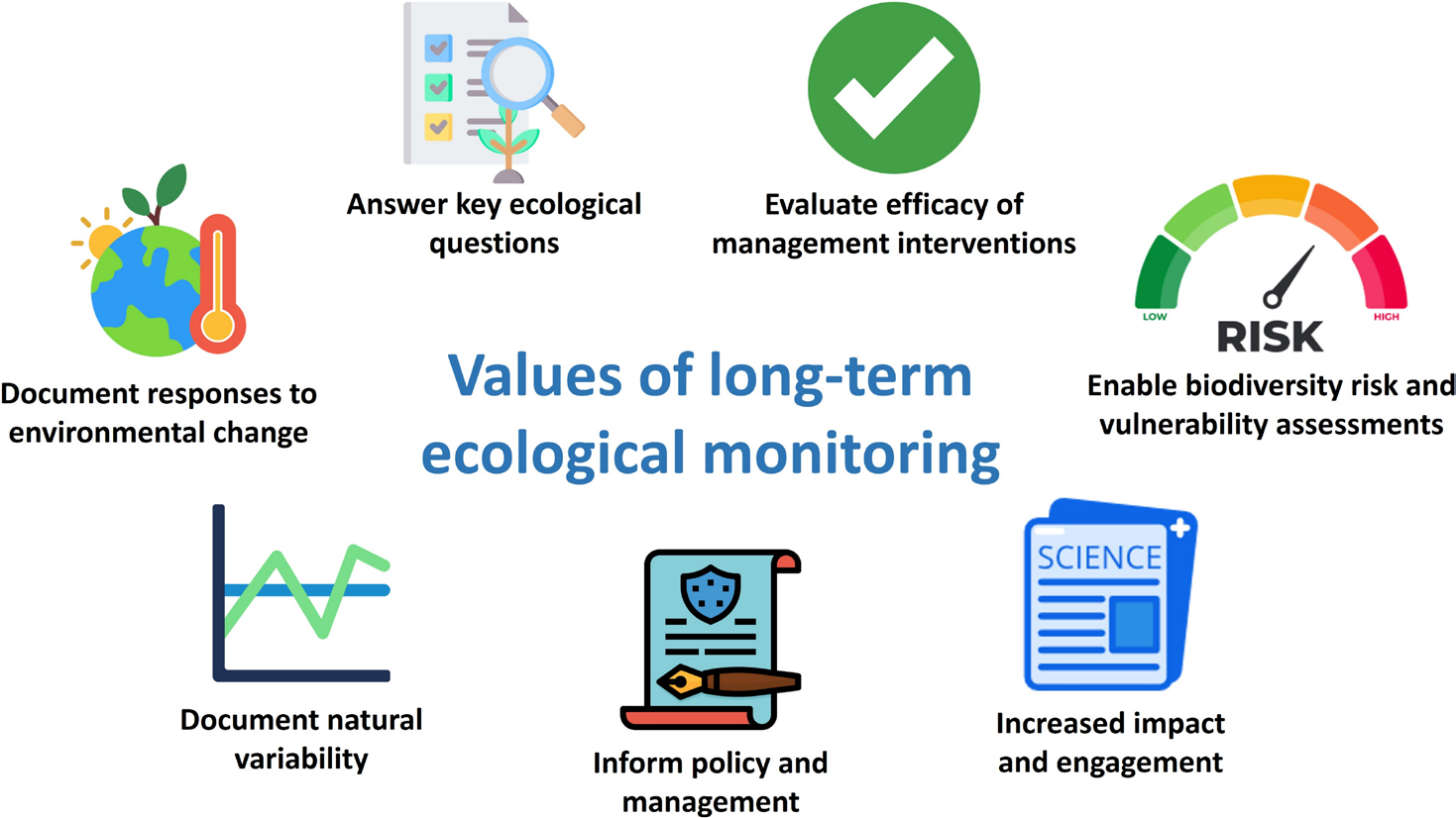
Key benefits of long‐term monitoring of ecosystems.

Long‐term monitoring (LTM) has long been hailed as a critical component of ecological research (Figure 1). It is typically defined as large‐scale and long duration, with the spatial and temporal scales of monitoring largely dependent on the organisms or ecosystems of interest. Ideally, LTM involves landscape scale or regional scale studies spanning 10 or more years. Studying interactions between biodiversity and environmental dynamics requires long‐term data collection to capture natural variability and cycles, and responses. LTM enables researchers to identify trends and distinguish between short‐term fluctuations, e.g., changes over hours or throughout a season, and long‐term shifts over years (Banyard et al. 2024; Bergstrom et al. 2021; Kubiszewski et al. 2024; Siegert et al. 2023; Wienecke et al. 2024).

Detecting change in biological communities over time has become increasingly important as we try to document and predict how ecosystems may respond to a growing number of key threats (Malhi et al. 2020). In the face of current global‐scale changes such as natural and human‐induced climate change and environmental modifications, including local‐scale human disturbances such as infrastructure development and pollution, which can further drive changes in natural communities (Brooks, Jabour, et al. 2019), the value of LTM studies is central in discerning natural variability from external driving forces (Magurran et al. 2010).

Results from long‐term studies are known to have a greater research impact than short‐term studies, with LTM papers typically having higher citation rates and their findings significantly influencing policy development and management practices (Hughes et al. 2017). Evaluating LTM studies in highly conserved regions (or regions of high conservation status and environmental importance) such as Antarctica, which are unique, highly sensitive, and undergoing rapid environmental changes (Chown et al. 2022; Hughes et al. 2021; Robinson 2022) is helpful when informing management decisions and biodiversity protection.

Monitoring Antarctic organisms over multiple years enables researchers to evaluate how Antarctic ecosystems are responding and changing over time. Antarctic organisms are experiencing physical and biological threats to their survival, including from environmental changes, introduced species, pollution, and disturbance. Due to the significant regional variation, robust baseline data and site surveys are essential for the purpose of detecting change across the different bioregions of Antarctica.

This review firstly highlights several key pressures on Antarctic ecosystems and secondly synthesizes LTM studies on nearshore marine and terrestrial biota in Antarctica. It also investigates which species, sites, biological parameters, and environmental factors have been and are being monitored long‐term. Long‐term changes in the biology of nearshore marine and terrestrial biota can inform management practices on species, communities, and ecosystems which are vulnerable or at risk—essential information for managers and policymakers.

### 1.1 Changing Antarctic Environments

Globally, human activities are causing rapid and unprecedented levels of ecological modification and damage. Polar regions are projected to be especially impacted due to their enhanced sensitivity to regional climate change (Constable et al. 2022; Goosse et al. 2018) and resident species' limited capacity for adaptation or migration (Hayward et al. 2024; Peck 2018; Peck et al. 2014; Pecl et al. 2017; Robinson et al. 2009). Several factors interact to influence Antarctica's weather patterns and extreme events, including the atmosphere, cryosphere, biosphere, ocean circulation, and topography (Siegert et al. 2023). As a result, climate change itself is increasingly complex in Antarctica, and so are its impacts locally, regionally, and globally (Chown et al. 2022).

Warming on land and in the oceans, changes in water availability on land, declines in sea ice, and increased ultraviolet radiation are only a few examples of climate change threats to Antarctic marine and terrestrial ecosystems. Both the Arctic and Antarctic regions have experienced some of the fastest warming on record (Anisimov et al. 2007; Siegert et al. 2023). For Antarctica, this warming was highly regional, and for a period, sea ice actually increased (Anisimov et al. 2007; Constable et al. 2022; Turner et al. 2016; Vaughan et al. 2003). More recently, however, warming has been documented across the continent (Chown et al. 2022) and temperature anomalies, such as heatwaves, have been reported (Siegert et al. 2023; Wille et al. 2024a; Wille et al. 2024b). The Southern Ocean is warming, threatening Antarctic ice sheets and causing sea ice to decline precipitously in recent years (Fretwell et al. 2023; Hobbs et al. 2024; Purich and Doddridge 2023). In addition, Antarctic biota is exposed to high ultraviolet radiation and windier conditions as a result of ozone depletion (Barnes et al. 2023; Burritt and Lamare 2016; Robinson et al. 2024), and is threatened by emerging diseases such as avian influenza (Banyard et al. 2024). As extreme events and changes in weather patterns intensify in strength and frequency, the impacts of other anthropogenic activities in Antarctica are expected to worsen (Abram et al. in press; Chown et al. 2022).

Despite its geographical isolation, direct and indirect human impacts on Antarctica have also been growing (Brooks, Jabour, et al. 2019; Brooks, Tejedo, and O'Neill 2019; Conroy 2023). The increasing human footprint on the Antarctic has introduced additional pressures to the extant ecological communities, namely biological invasions (Hughes, Pertierra, et al. 2015; McCarthy et al. 2019), contaminants (Bargagli 2008), and direct human disturbance because of infrastructure development, research, resource extraction, and tourism (Brooks, Jabour, et al. 2019).

### 1.2 Antarctic Biodiversity

Antarctica is an ecologically unique continent. Its geographical isolation and climate extremes have shaped unique assemblages of flora and fauna with a high level of endemism (Griffiths et al. 2009; Lebre et al. 2023; Mashamaite et al. 2023; Verleyen et al. 2021; Vyverman et al. 2010). Terrestrial biodiversity is largely limited to coastal and inland ice‐free havens (Convey et al. 2014; Lee et al. 2017; Terauds and Lee 2016). Maritime Antarctica, which includes the Western Antarctic Peninsula and the adjacent Scotia Arc archipelagos south of 60° S, has the highest levels of terrestrial biodiversity of anywhere in Antarctica, owing to the region's relatively mild climate (Convey et al. 2014). Two native and one invasive flowering plant species are restricted to the Maritime Antarctic region, which is also very favorable for non‐vascular plants (Convey et al. 2014; Molina‐Montenegro et al. 2019). Elsewhere, Antarctica's harsh climate of subzero temperatures, low bioavailable water, intense winds, and regular cycles of freeze–thaw and desiccation–rehydration largely restrict the permanent residents of the continent to smaller, resilient organisms, such as bryophytes, lichens, and invertebrates, in addition to microbes, fungi, and algae (Convey et al. 2014; Mashamaite et al. 2023). These organisms are the extremophiles of Antarctica's biodiversity, remaining on land or in the surrounding shallow waters of the continent year‐round, and being exposed to the full climate ranges of the Antarctic environment. Larger marine mammals and birds live in Antarctic waters and coastal zones for the summer breeding season, migrating to warmer sub‐Antarctic or temperate waters during winter, except for Emperor ( *Aptenodytes forsteri* ) and Adélie ( *Pygoscelis adeliae* ) penguins, which inhabit the Antarctic year‐round.

Nearshore marine zones (less than 100 m in depth) are dominated by invertebrate and macroalgae communities (Peck 2018). While the deeper waters of the Southern Ocean have been investigated and studied since as early as the 1900s (Fogg 2005), nearshore marine zones, in contrast to intertidal and shallow‐water studies globally, remain some of the most understudied ecosystems in Antarctica (Griffiths and Waller 2016). These shallow marine ecosystems are highly diverse and show great levels of regional specificity, owing to limited connectivity around the continent (Baird et al. 2012; Lau et al. 2023). This places these communities at elevated risk of anthropogenic threats as migration, colonization, and recruitment are locally restricted (Clark et al. 2015; Griffiths et al. 2024; Lau et al. 2023; Moon et al. 2017; Peck 2018).

Antarctic flora and fauna have long been regarded as having intrinsic value with recognized conservation significance. The first measures of protection were adopted in 1964 within the Antarctic Treaty (ATCM 1964). Environmental protection was later reinforced by the 1991 Protocol on Environmental Protection to the Antarctic Treaty (ATCM 1991), which designates Antarctica as a “natural reserve, devoted to peace and science” (Article 2) and sets basic principles applicable to human activities in Antarctica (Article 3). Annex V to the Environmental Protocol pertains to designation of protected areas [Antarctic Specially Protected Areas (ASPA) and Antarctic Specially Managed Areas (ASMA)]. Biodiversity protection is essential in maintaining healthy populations and communities. Scientific guidance on regions that are already, or are likely to change, is required to inform placement of protected regions (Lee, Terauds, et al. 2022).

Terrestrial ecological communities have been classified into 16 biologically distinct Antarctic Conservation Biogeographic Regions (ACBR) across the Antarctic continent (Terauds et al. 2012; Terauds and Lee 2016). While these ecoregions are considered to be of high conservation value, they are not adequately protected. Less than 0.5% of vegetation biodiversity within ACBRs falls within the protected areas network (Anderson et al. 2024; Hughes et al. 2016) inside ASPAs. ASPAs are biased towards more charismatic species: marine mammals, seabirds, and penguins, although a recent study suggests that mosses are also reasonably well represented within them (Anderson et al. 2024). While 44% of species occur within one or more ASPAs overall, this includes ASPAs not designated for species or community conservation (Wauchope et al. 2019). This percentage also does not include any nearshore marine species, microbes, or fungi that fall within ASPA zones (Wauchope et al. 2019). Antarctic microbial communities in particular are not well protected within ASPAs (Hughes, Cowan, and Wilmotte 2015).

**FIGURE 2:**
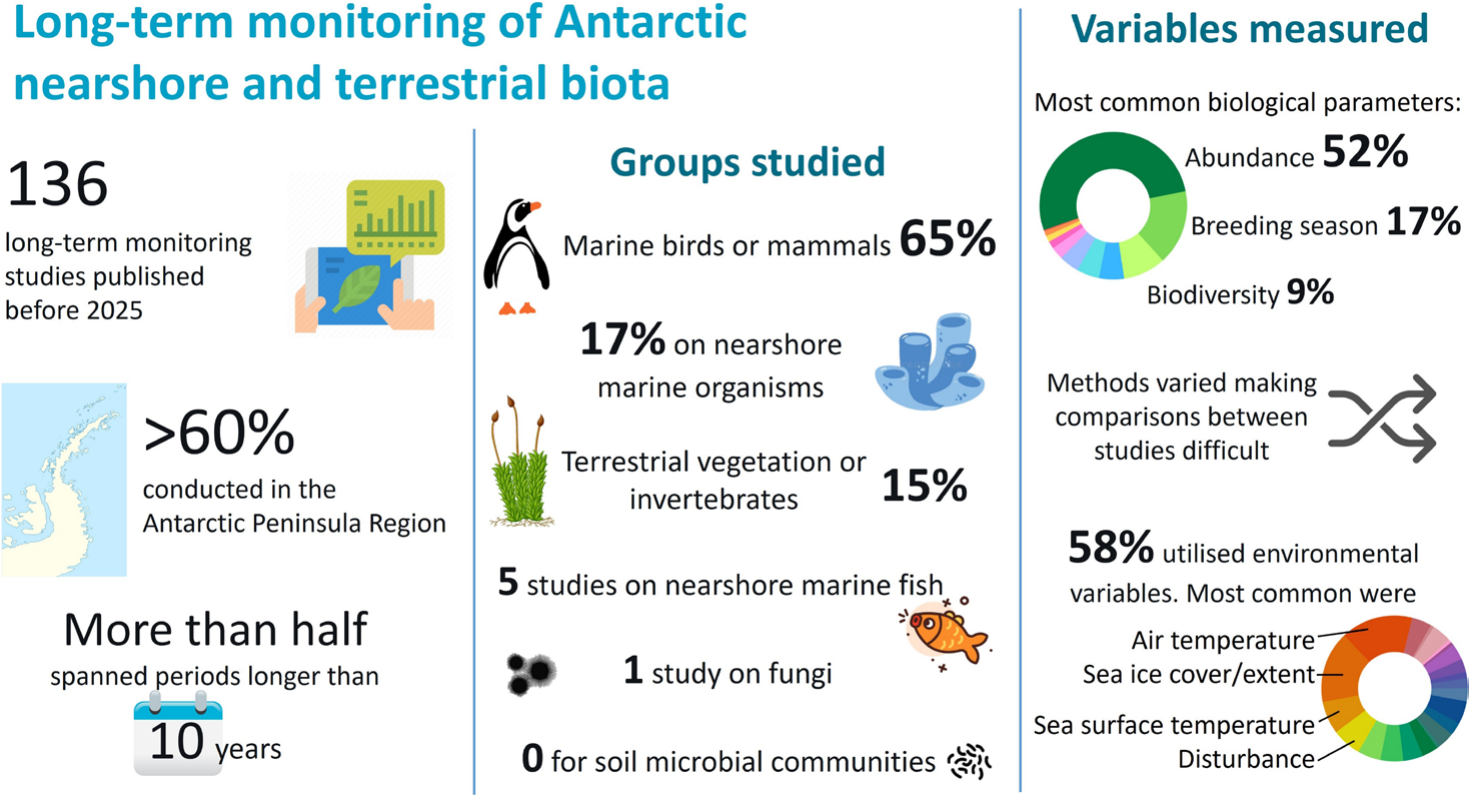
Summary of published long‐term monitoring studies in Antarctic nearshore and terrestrial ecosystems. Note that there are several Antarctic biota groups and locations that are understudied. More than half the studies measured both environmental co‐variates and biological parameters.

Marine ecosystems are not incorporated or represented in ACBRs and are under‐protected (Scott 2013). Marine ecoregions have been proposed as a way of classification (Brooks et al. 2020; Koubbi et al. 2016; Koubbi et al. 2010), but these have not yet been used as a guide for conservation or protection. The Antarctic Treaty and the Convention on the Conservation of Antarctic Marine Living Resources (CCAMLR) provide broad‐scale protection to the surrounding waters of Antarctica (Nyman 2018). Regions designated as marine protected areas (MPAs) and marine ASPAs can offer higher levels of protection, such as enforcing no‐take restrictions on fishing and many other activities, but are currently limited in their coverage of nearshore marine zones. For example, the only continental Antarctica nearshore marine areas currently protected by MPAs are in the Ross Sea (Brooks et al. 2020).

The rapidly changing Antarctic environment and the increase in the number of areas adversely impacted by human activities highlight the need for ecological monitoring of these insufficiently protected ecosystems (Hughes et al. 2016; Leihy et al. 2020; Wauchope et al. 2019). Rapid warming, glacial retreat, sea ice decline, ice sheet mass loss, and ocean acidification are only some examples of rapid climate change occurring in Antarctica (Chown et al. 2022 and references within; Hobbs et al. 2024; Purich and Doddridge 2023; Robinson et al. 2023; Siegert et al. 2023). Existing conservation strategies and resources are inadequate under future climate scenarios, especially for Antarctic terrestrial biodiversity (Lee, Terauds, et al. 2022). Detecting responses in biota to the changing environment will inform conservation decisions and priorities (Lee, Terauds, et al. 2022). Therefore, LTM of biota contributes to the information decision makers need to tailor conservation efforts and resources to species, location, and predicted changes in climate. However, to successfully monitor change across Antarctic ecosystems and to highlight those most at risk requires consistent standards of data collection that enable comparison across regions, communities, and species.

Here, we review the challenges facing Antarctic biodiversity and evaluate the current state of biological monitoring in Antarctica to (1) synthesize existing LTM studies and programs and (2) assess how LTM can better support conservation and management of Antarctica's biodiversity in the face of profound environmental change.

## 2 Key Pressures on Antarctic Biodiversity

There are numerous challenges that biota face living in Antarctica and changes in the environment to which they are adapted could threaten their existence. In this section, we summarize some of the key drivers of biodiversity loss including climate change, invasive species, ocean acidification, changing sea ice dynamics, physical disturbance, and pollution.

### 2.1 Anthropogenic Warming

Anthropogenic climate change is the greatest challenge species and communities are facing this century (e.g., Gutt et al. 2021). As concentrations of atmospheric greenhouse gases continue to climb, communities in polar regions are predicted to be the worst impacted by temperature increases (Chown et al. 2022; Goosse et al. 2018) as well as extreme weather events (Siegert et al. 2023). Western Antarctica has already experienced rapid warming trends (Znój et al. 2017), exceeding 1.5°C on the Western Antarctic Peninsula (Turner et al. 2005), and 1°C in nearshore coastal waters (Jun et al. 2020; Meredith and King 2005). This region is projected to experience further temperature increases despite a two‐decade‐long pause in warming during the 21st century (Chown et al. 2022; Turner et al. 2020).

The increases in temperature in West Antarctica have led to large‐scale and rapid glacial retreat (Cook et al. 2016; Milillo et al. 2022), shifts in sea ice regimes (Hobbs et al. 2024; Purich and Doddridge 2023; Wille et al. 2024a; Wille et al. 2024b) and an increase in the incidents of extreme weather and anomalous events (Abram et al. in press; Purich and Doddridge 2023; Robinson 2022; Robinson, Klekociuk, et al. 2020; Siegert et al. 2023). On land, water availability, a major driving factor of continental/terrestrial community structure, is modifying species' distributions and community structure (Convey and Peck 2019; Lee, Waterman, et al. 2022). Where it becomes warmer and wetter, vegetation is expanding (Cannone et al. 2022; Prather et al. 2019). Where it becomes drier, vegetation health declines, species composition is altered, and rapid ecosystem collapse can occur (Bergstrom et al. 2015; Bergstrom et al. 2021; Lee, Waterman, et al. 2022; Robinson et al. 2018).

East Antarctica has yet to experience the long‐term warming trends observed in the west of the continent (Robinson et al. 2018). This is largely attributed to the effects of the ozone hole, which has developed above the continent every spring since the 1970s (Robinson and Erickson III 2015). Ozone depletion has promoted a more positive Southern Annular Mode (SAM) causing stronger Jetstream winds over the Southern Ocean (Abram et al. 2014), thereby reducing the mixing of warmer tropical air by restricting the equatorward movement of the Southern Ocean Westerlies (Robinson and Erickson III 2015). However, recent breakdowns of this phenomenon saw the first recorded heatwaves around Antarctica in the 2019/2020 spring–summer season (Robinson, Klekociuk, et al. 2020). An even more extreme heat anomaly occurred in March 2022, when an atmospheric river delivered subtropical heat and moisture to Antarctica (Wille et al. 2024a, 2024b). Although Continental Antarctica has not yet seen prolonged large‐scale temperature increases, changes in wind patterns and precipitation events have already been shown to impact ecological communities (Bergstrom et al. 2021; Robinson et al. 2018).

### 2.2 Changing Sea Ice Dynamics

Climate indices have been shown to have a strong influence on sea ice conditions and thus on coastal marine communities (e.g., Fretwell et al. 2023; Lohrer et al. 2021; Palmer et al. 2021). Additionally, in recent years, winter sea ice conditions have been unusual in the southern Ross Sea (e.g., Leonard et al. 2021; Robinson et al. 2023) and the entire continent experienced unprecedented sea ice extent minima in 2023 and 2024 (Abram et al. in press; Purich and Doddridge 2023; Turner et al. 2022) due to oceanic warming.

Sea ice dynamics play a major role in structuring marine benthic communities (Amsler et al. 2023; Clark et al. 2013; Griffiths et al. 2024). Warmer sea and air temperatures are altering seasonal ice melt patterns, with summer ice melt occurring sooner and winter ice formation occurring later (Hobbs et al. 2016). Both fast ice and sea ice have shown dramatic declines in recent years (Hobbs et al. 2024; Purich and Doddridge 2023). As shifts in sea ice regimes occur, nearshore communities are being exposed to higher levels of sunlight (and UV radiation, Robinson et al. 2024), increasing primary production, altering community function and, in some cases, causing major shifts in benthic community composition (e.g., from invertebrate to macroalgae‐dominated communities in Clark et al. 2013). Currently, limits on seasonal sunlight regulate the interactions between algae and invertebrates (Mills et al. 2025). As these communities begin to shift from heterotrophic to autotrophic states, and tipping points are encroached (Scheffer et al. 2001), up to one third of shallow marine species could become locally extinct (Clark et al. 2013).

Further, the survival of several sea‐ice‐dependent species, such as Weddell seals and Emperor and Adélie penguins, is at risk due to rapid shifts in sea ice regimes (Dunn et al. 2025; Fretwell et al. 2023; LaRue et al. 2019). Already, Emperor penguin populations have reduced significantly and rapidly in response to sea ice loss, reducing breeding and foraging success, and are thus at risk of extinction (Fretwell et al. 2023; Lee, Terauds, et al. 2022; Wienecke et al. 2024).

### 2.3 Ocean Acidification

Like the rapid warming of polar zones, ocean acidification is occurring at an accelerated rate in polar oceans (Hancock et al. 2020). As colder waters have a greater affinity for atmospheric gases, CO_2_ is dissolved more readily in polar waters (McNeil and Matear 2008). Ocean warming has implications for global overturning circulation which cycles heat, carbon, oxygen, and nutrients throughout the world oceans (Kubiszewski et al. 2024; Li et al. 2023). The Southern Ocean is at elevated risk for ocean acidification as its circumpolar current prevents the mixing of waters, increasing the concentration of CO_2_ and reducing the introduction of warmer waters (McNeil and Matear 2008). Thus, the responses of Southern Ocean ecological communities will prove seminal for predicting global responses to ocean acidification. Ocean acidification reduces concentrations of calcium carbonate and the acidity of seawater, and has been shown to be a major threat for some Antarctic species, and particularly for early life stages, although there is potential for acclimation in others (e.g., Figuerola et al. 2021; Peck 2018).

Krill ( *Euphausia superba* ) populations are predicted to be at high risk under projected ocean acidification models (Constable et al. 2014), warming oceans, and sea ice change (e.g., Kawaguchi et al. 2023). The loss of such a keystone species, the main prey of seals, penguins, and whales, could lead to the collapse of both deep and shallow ocean ecosystems (Flores et al. 2012; Kawaguchi et al. 2013). The flow‐on effects onto terrestrial zones are understood to a lesser extent, but given that terrestrial ecosystems rely on nutrients from seabird guano (Bokhorst et al. 2016; Yin et al. 2023) species' range shifts, population growth and declines, and modified food chains would likely have implications for on‐land communities.

### 2.4 Invasive Species

Another significant pressure on global biodiversity is biological invasions (Chown et al. 2022; Early et al. 2016; Leihy et al. 2023). Invasive non‐native species can modify competition within communities and can outcompete native species for food, water, sunlight, and space. In this instance, native species become displaced, resulting in a decline in their abundance and health, and reduced overall ecosystem diversity (Hughes et al. 2020). For example in Maritime Antarctica, the invasive grass *Poa annua* has been demonstrated to outcompete the only two native vascular species, *Deschampsia antarctica* and *Colobanthus quitensis*, reducing their overall abundance; an effect amplified by warming temperatures (Cavieres et al. 2018). As temperatures across Antarctica increase, the potential for further invasions may grow owing to increased habitat suitability for non‐natives (Bokhorst et al. 2024). Changes in food web dynamics, such as the introduction of a predator or grazer, can have cascading effects on native species' survival and ecosystem functioning (see review Convey and Peck 2019). Once invasive species establish, habitat degradation can further disrupt ecosystem function. The risk of invasion by *Membranipora membranacea* could result in the loss of Antarctic kelp forests as the invasive bryozoan weakens kelp structures and makes them more prone to breakage (Avila et al. 2020; Krumhansl et al. 2011).

The risk of invasion in Antarctica has grown with increased human activity and modified environments (Brooks, Tejedo, and O'Neill 2019; Pertierra, Hughes, et al. 2017), acting as vector pathways and creating ameliorated conditions for non‐native species (Frenot et al. 2005; Hughes et al. 2020; McCarthy et al. 2019). However, increased biosecurity appears to be slowing introductions of non‐native species into terrestrial Antarctica, with rates of introductions stabilizing or slowing in recent years (Leihy et al. 2025). But unless human activities, numbers and intensities of visits, and changes in the region's climate all slow, stricter biosecurity measures will need to be implemented (Leihy et al. 2025). This is in addition to assessment and management of ongoing risks of non‐natives establishing in Antarctica (Onley et al. 2024). Visitation by researchers and tourists is the most significant pathway for non‐native propagules to enter the region, with the potential for more than 70,000 viable exotic seeds to be brought into the region annually (Huiskes et al. 2014). Transport of viable propagules and living individuals of alien plant species, invertebrates, fungi, and bacteria has been detected in cargo shipments of equipment and food being transported into research stations (Houghton et al. 2016; Hughes et al. 2010; Hughes et al. 2011; Hughes et al. 2020; Onley et al. 2024). There is a heightened risk of research stations being colonized by introduced synanthropic species, such as *P. annua* or the exotic midge *Psychoda albipennis* (Hernandez‐Martelo et al. 2024; Molina‐Montenegro et al. 2019; Molina‐Montenegro et al. 2014; Pertierra, Aragón, et al. 2017). Many research stations lack adequate biosecurity measures and monitoring of potential invasions (Hughes and Pertierra 2016). In the regions with the largest human footprint, principally the Antarctic Peninsula, climate change and habitat modification add to this risk of invasion (Frenot et al. 2005; Hughes et al. 2020). Given its increasingly milder conditions, closer proximity to other continents, and the level of human disturbance and visitation, the Antarctic Peninsula is the region at greatest risk of invasion (e.g., Chown et al. 2022; Dawson et al. 2024; Duffy et al. 2017).

In marine environments, bioinvasion can occur by transportation on ships as well as with currents and wind‐driven transport. Invasive species and their propagules can be transported via vessels (biofouling on hulls or in ballast water) or with floating and drifting objects (e.g., rafting on ocean debris or kelps), providing consistent pathways for their introduction into Antarctic waters (Avila et al. 2020; Dawson et al. 2024; Fraser et al. 2022; Fraser et al. 2018; Hughes and Ashton 2017). Non‐native species hitchhiking on vessel hulls can travel considerable distances from outside and within Antarctic waters (Hughes and Ashton 2017; McCarthy et al. 2022). With warming oceans and increased marine traffic, the likelihood of eventual establishment of such invasive species is growing (López‐Farrán et al. 2021).

### 2.5 Human Disturbance

As the human footprint (research, fishing, infrastructure, and tourism) in Antarctica has continued to climb (Brooks, Jabour, et al. 2019; Huiskes et al. 2014; IAATO 2024a), the impact on Antarctica's biodiversity remains vastly under‐monitored. Studies which have investigated the impact of this growing footprint have highlighted the changes in ecological communities in response to direct human activities, such as contaminants and physical disturbance (see reviews Bargagli and Rota 2024; Brooks, Tejedo, and O'Neill 2019). Owing to its isolation, public perception of a pristine and preserved Antarctica persists (Hughes et al. 2013; Leihy et al. 2020). However, many of the continent's species and communities are under threat due to both direct and indirect human activities. While research on the frozen continent provides essential ecological insights, disturbance from research sampling, trampling, and especially research station infrastructure has large, and in many instances unknown, implications on the surrounding biodiversity (Brooks et al. 2024; Leihy et al. 2020). Ecosystem structure and function can be altered in response to this direct human disturbance (Ayres et al. 2008; Brooks, Jabour, et al. 2019), although the broad‐scale effects are not fully known.

The Antarctic Peninsula region has the highest potential for direct human disturbance. Its close proximity to South America and more moderate climate compared to elsewhere in Antarctica make it an appealing region for research and tourism. Of the 94 historic and 81 current research stations in the Antarctic, almost half are on the Antarctic Peninsula and adjacent islands (Brooks, Jabour, et al. 2019; COMNAP 2025).

The infrastructure of Antarctic stations covers a total area of 0.393 km^2^ (with a total 5.242‐km^2^ human disturbance footprint within ice‐free areas). Although this is remarkably small considering the size of the Antarctic continent, 81% of these stations are built on the extremely small portion (0.44%) of Antarctica that is ice‐free and thus is potentially important habitat for terrestrial biota (Brooks, Jabour, et al. 2019). Three‐quarters of these buildings exist in accessible ice‐free areas that are within 5 km of the coast. Additionally, Antarctica is seeing an upward trend in tourism rates, with more than 122,000 seaborne tourists visiting the continent in the 2023/24 summer season; more than double the 55,000 tourists who visited Antarctica in 2018/19 (IAATO 2024b). Similarly, the number of these passengers landing in Antarctica has almost doubled over this same period, from 45,000 to 79,000, with a 1.4‐fold increase in the number of landing sites (IAATO 2024a, 2024b). Growth in visitation and especially landing rates leads to increased propagule pressure, trampling, and pollution (Huiskes et al. 2014).

Continued disturbance from commercial exploitation in the Southern Ocean also places pressure on marine ecosystems. The heavy extraction of marine mammalian predators in the 19th century is thought to have substantially altered the marine ecosystem (Hofman 2019), although the absence of long‐term observations makes the extent of ecological damage mostly unknown. Existing fisheries, managed by CCAMLR, operate in Antarctic waters (Brooks 2013). Krill, fish, and squid are extracted, with pushes to increase current allowable catches (Brooks 2013).

### 2.6 Pollution

Through countless scientific and non‐scientific expeditions, long‐term human presence in Antarctica has left an undesirable legacy of pollution and chemical contaminants (Bargagli 2008). Prior to the inception of the Madrid Protocol, research stations and their surroundings had been the sites for chemical and industrial waste dumping. Although levels of contaminants are typically considered to be lower than elsewhere in the world, crude oil spills, heavy metal accumulation, human waste, and pollutant and plastics runoff have all become prevalent in Antarctic ecosystems (Aves et al. 2022; Bargagli 2008). Contaminants can reduce species diversity or completely exclude taxa from polluted areas (Koppel et al. 2021; Palmer et al. 2021; Polmear et al. 2015; Powell et al. 2003).

The management and remediation of polluted or dump sites has had success in the past, including site recovery attempts at some research stations, such as Casey (Snape et al. 2001), although the long‐term recovery of these affected communities has yet to be reported on. In addition, climate warming and permafrost melting threaten to release frozen contaminants and make them bioavailable (Kubiszewski et al. 2024). Tighter regulations around waste management in Antarctica were introduced in 1991 (ATCM 1991), although the continued presence of humans in Antarctica perpetuates the risk of introducing foreign substances into the environment.

Manipulative experiments provide valuable insights into potential responses and resilience of communities to major climate‐associated drivers such as warming, sea ice dynamics, ocean acidification, invasive species, disturbance, and pollution (e.g., Ashton et al. 2017; Cummings et al. 2019; Stark et al. 2019). However, there is a need for biological and environmental monitoring under natural conditions in the field to determine how communities are currently responding to amplified environmental stressors (e.g., Griffiths et al. 2024).

## 3 State of Long‐Term Biological Monitoring in Antarctica

### 3.1 Literature Search

To gain insight into existing LTM of nearshore and terrestrial biological systems in Antarctica, we conducted an extensive search using SCOPUS for papers published in English before 2025 featuring several keywords (including Antarctic*, bio*, long‐term, monitor*; see supporting information for a detailed list). Searches for specific Antarctic taxa and species were also undertaken. Metadata for articles that included any or a combination of these keywords in their title, abstract, or keyword list were downloaded. Articles from this list were excluded if it was clear in the title or abstract that the study did not (1) occur in the area of Antarctic Treaty governance including Maritime or Continental Antarctica, (2) occur in nearshore marine (< 100 m depth) or terrestrial zones, (3) involve biota, (4) measure a biological parameter, (5) occur over the long term, or (6) represent unmanipulated variation. We define monitoring studies as long term if they had at least three or more monitoring time points over at least three years, including non‐consecutive seasons (i.e., studies reporting non‐annual data, including biannual, decadal or irregular time points). Remaining papers were closely examined and were included in this review if they met criteria 1–6 above. Studies with experimental and manipulative treatments were not considered unless papers reported non‐manipulated controls.

We widened our search to capture papers in preparation as well as studies in other languages by contacting various Antarctic scientific groups including Scientific Committee on Antarctic Research Expert Groups and Scientific Research Programs (e.g., ANTOS, AnT‐ERA, and AntEco) via email distribution lists, however, this resulted in very few responses. We also extended our search to National Antarctic Data Centers for publications that met criteria 1–6, which also added few papers that the SCOPUS search did not capture. Further, we acknowledge the limitation of the “online‐only” approach in capturing earlier publications not discoverable online, as well as the potential exclusion of studies published in other languages.

### 3.2 Evaluation of Published LTM Studies

Each study was examined to determine the locations, biota, and biological parameters that were monitored. First, we evaluated the spatial distribution of the area where the studies took place. Second, Antarctic species were categorized into the following groups: marine birds and mammals, marine fauna, vegetation, terrestrial invertebrates, microbes, and algae and fungi. Third, for each group, we evaluated the biological characteristics that were monitored. These included abundance, biodiversity, breeding season, diet, survival, growth, physiology, behavior, distribution, competition, and phenology.

Summary statistics of biota groups, or biological characteristics, were then generated for discussion. Studies which monitored more than one species/group or measured more than one characteristic were therefore included more than once. We also documented the environmental parameters recorded in those studies and any trends indicated. The compiled dataset is publicly available at https://doi.org/10.26179/nvjw‐qf32 (Waterman et al. 2025).

### 3.3 LTM Studies in Nearshore and Terrestrial Antarctica

The number of long‐term biological monitoring studies discovered was limited, with a total of 136 publications. Despite this relatively low number, more than half of these publications had data series spanning longer than 10 years (Figure 2). Less than 20% had data collections over a period less than 5 years. Across the reviewed studies, changes in local and regional species' abundances, distributions, and/or functions are reported for most biota groups, separated according to terrestrial ecosystems (Table 1), marine birds and mammals (Table 2), and nearshore biota (Table 3). Below, we describe these main findings with respect to the spatial distribution of studies, the biota groups, and biological parameters included in LTM and the associated environmental monitoring. Finally, we draw on these findings, which are summarized in Figure 2, to advise future LTM data collections with recommendations for systematic and harmonized research efforts.

**TABLE 1:** Long‐term monitoring studies of terrestrial biota in Antarctic Maritime and Continental regions.

Functional group	Species/taxa	Location	Declared ASPA	Study duration	Study years	Environmental parameters	Results/biological response	References
**Terrestrial Invertebrates**	Arthropods	South Orkney Islands		1984–1995	Yearly	↑Air temperature (summer), ns Radiation, ns Rainfall	ns Body water	Convey et al. (2003)
	Invertebrates	Victoria Land		1993–2017	Opportunistically	ns Soil moisture; ns Soil conductivity; ns pH	↓Abundance, ns Biodiversity	Andriuzzi et al. (2018)
	Micro‐invertebrates	Dronning Maud Land		1991–2002	1991/92, 1993/94, 1996/97, 2001/02	/	ns Abundance, ns Biodiversity	Sohlenius and Boström (2005)
	Nematode spp.	West Antarctic Peninsula		2007–2010	2007, 2008, 2010	ns Moss temperature, ns Moss moisture	ns Abundance	Newsham et al. (2021)
	Nematode spp. *Scottnema lindsayae, Eudorylaimus antarcticus*, *Plectus antarcticus*	Victoria Land		1993–1999	1993, 1995, 1999	*Soil moisture	*Abundance, *Life cycle	Porazinska et al. (2002)
	*Scottnema lindsayae*			1993–2005	Yearly	↓Soil temperature	↓Abundance	Barrett et al. (2008)+
	*Scottnema* spp. *Eudorylaimus* spp.			1993–2013	Yearly	↓Then ns Air temperature, ↑↓Lake ice thickness, ↑ns Solar radiation, ↓↑ Annual stream flow rates (shifts in 2002)	↓Then ns Abundance (shift in 2002)	Gooseff et al. (2017)+
	Non‐native Invertebrates	Victoria Land		2006–2016	Yearly	/	↑Abundance, ↑Biodiversity	Newman et al. (2018)
**Vascular vegetation**	Antarctic Hair Grass— *Deschampsia antarctica*	North‐West Antarctic Peninsula		1964–1990	1963/64, 1967, 1974, 1977, 1981, 1990	↑Air temperature	↑Abundance, ns Physiology	Fowbert and Lewis Smith (1994)+
				1964–2008	1964, 1967, 1974, 1977, 1981, 1990, 2006/7, 2007/8	/	ns Abundance	Parnikoza et al. (2009)+
				1972–2020	1997/8, 2003/4, 2013/4 and historical photos from 1972 to 2020	ns Moisture, ns Soil properties, ns Human disturbance, ns Erosion	ns Abundance	Putzke et al. (2022)
			128; 151	1991–2009	Yearly	Plant succession following glacial retreat	↑Abundance	Olech (2010)
		South Orkney Islands		1960–2018	1960, 2009, 2018	↑Air temperature, ↑Animal disturbance, ↑Deglaciation	↑Abundance	Cannone et al. (2022)
				1965–1992	1965, 1967, 1977, 1985, 1992	↑Mean summer temperature	↑Abundance, ↑Seedling survival	Lewis Smith (1994)

				2009–2011	Yearly	ns Timing of snow melt	ns Distribution	Park et al. (2012)
	Antarctic Pearlwort—*Colobanthus quitensis*	North‐West Antarctic Peninsula		1964–1990	1963/64, 1967, 1974, 1977, 1981, 1990	↑Air Temperature	↑Abundance, ns Physiology	Fowbert and Lewis Smith (1994)+
				1964–2008	1964, 1967, 1974, 1977, 1981, 1990, 2006/7, 2007/8	/	ns Abundance	Parnikoza et al. (2009)+
				1972–2020	1997/8, 2003/4, 2013/4 and historical photos from 1972 to 2020	ns Moisture, ns Soil properties, ns Human disturbance, ns Erosion	ns Abundance	Putzke et al. (2022)
			128; 151	1991–2009	Yearly	Plant succession following glacial retreat	↑Abundance	Olech (2010)
		South Orkney Islands		1960–2018	1960, 2009, 2018	↑Air temperature, ↑Animal disturbance, ↑Deglaciation	↑Abundance	Cannone et al. (2022)
				1965–1992	1965, 1967, 1977, 1985, 1992	↑Mean temperature (summer)	↑Abundance, ↑Seedling survival	Lewis Smith (1994)
*Poa annua* (introduced)	South Shetland Islands	128; 151	1991–2009	Yearly	Plant succession following glacial retreat	↑Abundance	Olech (2010)
**Non‐Vascular vegetation**	Lichen spp. *Usnea aurantiaco‐atra*	North‐West Antarctic Peninsula	151	1988–2008	1988, 1990, 2007–2008	Reponses to glacial retreat	↑↓Abundance	Olech and Słaby (2016)
			128; 151	1991–2009	Yearly	Plant succession following glacial retreat	↓Biodiversity, ↑↓Abundance	Olech (2010)
				2009–2014	2009–2011, 2014	/	ns Productivity	Beltrán‐Sanz et al. (2022)
	*Acarospora macrocyclos*			1991–2015	1991, 2002, 2015	ns Temperature, Response to glacial retreat	↑↓Growth rate	Sancho et al. (2017)
	*Bellemerea* sp.			1991–2015	1991, 2002, 2015	ns Temperature, Response to glacial retreat	↑↓Growth rate	Sancho et al. (2017)
	*Buellia latemarginata*			1991–2015	1991, 2002, 2015	ns Temperature, Response to glacial retreat	ns Growth rate	Sancho et al. (2017)
	*Caloplaca sublobulata*			1991–2015	1991, 2002, 2015	ns Temperature, Response to glacial retreat	↑↓Growth rate	Sancho et al. (2017)
	*Rhizocarpon geographicum*			1991–2015	1991, 2002, 2015	ns Temperature, Response to glacial retreat	↑↓Growth rate	Sancho et al. (2017)

	*Usnea antarctica*			1991–2015	1991, 2002, 2015	ns Temperature, Response to glacial retreat	↑↓Growth rate	Sancho et al. (2017)
	Lichen spp. predominantly *Caloplaca saxicola* and *Candelariella flava*	Victoria Land	106	1961–2018	1961, 2004, 2018	↑Air temperature, ns humidity	ns Abundance, ns Species composition	Colesie et al. (2022)
	Moss spp.	South Shetland Islands		1972–2020	1997, 2003, 2013 and 1972–2020 from historical photos	ns Moisture, ns Soil properties, ns Human disturbance, ns Erosion	↓Abundance, *Species composition	Putzke et al. (2022)
			128; 151	1991–2009	Yearly	Plant succession following glacial retreat	↓Biodiversity, ↓Abundance	Olech (2010)
	Moss spp. *Schistidium antarctici*, *Bryum pseudotriquetrum,* *Ceratodon purpureus*	Wilkes Land		1990–1995	1990–92, 1993–95	↓Air temperature, ↓Water availability	*Species composition	Melick and Seppelt (1997)
			135	2000–2013	2000, 2003, 2008, 2011–2013	↓Water availability, ↑Wind speed	*Species composition, *Physiology	Robinson et al. (2018)+
			135	2003–2013	2003, 2008, 2011–2014	/	↑↓Abundance (moss lichen cover), ↑↓Physiology	King et al. (2020)+
Moss spp. predominantly *Bryum argenteum var. muticum*	Victoria Land	106	1961–2018	1961, 2004, 2018	↑Air temperature, ns Humidity	↑Abundance, ns Species composition	Colesie et al. (2022)
**Algae/cyanobacteria**	*Prasiola crispa*, *Nostoc commune*	Victoria Land	106	1961–2018	1961, 2004, 2018	↑Air temperature, ns Humidity	ns Abundance, ns Species composition	Colesie et al. (2022)
	*Phormidium* spp. *Nostoc* spp.			1993–2013	Yearly	↓ then ns Air temperature, ↑↓Lake ice thickness, ↑ns Solar radiation, ↓↑Annual stream flow rates (shifts in 2002)	↓↑*Nostoc* Biomass ns *Phormidium* Biomass ↓↑Primary productivity (shifts in 2002)	Gooseff et al. (2017)
**Fungi**	Fungal spp.	South Shetland Islands	128; 151	1991–2009	Yearly	Plant succession following glacial retreat	↓Biodiversity, ↓Abundance	Olech (2010)

*Note:* All species are native to Antarctica unless indicated. Note that some studies predate ASPA designation.

Abbreviations: * = significant change, / = no measurement, + = studies that may overlap results within corresponding monitoring programs, ↓ = significant decrease, ↑ = significant increase, ns = non‐significant result.

**TABLE 2:** Long‐term monitoring studies of marine birds and mammals in Antarctic Maritime and Continental regions.

Functional group	Species	Location	Declared ASPA	Duration	Study years	Environmental parameters	Results/biological response	References
**Penguins**	Adelie Penguins— *Pygoscelis adeliae*	Adelie Land		1950–2004	Yearly	↑SAM, ↓SOI, ns Air Temperature, ↓MSA, ns SIL	*Breeding Season	Barbraud and Weimerskirch (2006)
				1979–2010	Non‐continuous (combined datasets)	/	↑Abundance	Southwell et al. (2015)
				1984–2000	Yearly	ns SST, ↓SIC, ↓SIE, *SOI	↑Abundance, ns Survival	Jenouvrier et al. (2006)
				1984–1999	Yearly	Impact of airstrip development	↑Abundance, ns Breeding success	Micol and Jouventin (2001)
		Dronning Maud Land		1961–2004	Sporadically	/	↑Abundance, ns Growth	Kato and Ropert‐Coudert (2006)
				1995–2000	Yearly	ns Water depth temperature, ns Conductivity	ns Behavior, ns Growth	Naito et al. (2002)
		Enderby Land		1979–2010	Non‐continuous (combined datasets)	/	↑Abundance	Southwell et al. (2015)
		Mac. Robertson Land		1990–1999	Yearly	ns SIE	ns Abundance, ns Breeding success, ns Growth, *Diet	Clarke et al. (2002)
				1990–2009	Yearly	ns SIC, ns SST, ns SIE, *SAM, ns SOI, ns ENSO, *Air temperature, *Wind speed, *Snow days, *Wind direction	ns Breeding success, *Phenology	Emmerson et al. (2011)
				1990–2020	Yearly	*Summer SIE, *SIC, ns SAM, ns SOI, ns Air temperature, ns Windchill	↓Abundance, ns Breeding success, ↓Survival, ns Growth	Emmerson and Southwell (2022)
				1991–2004	Yearly	ns Windchill	ns Abundance, ns Breeding Success	Smiley and Emmerson (2016)
				1991–2003	Yearly	/	ns Breeding Success, ns Diet	Tierney et al. (2009)
		Mawson		1979–2010	Non‐continuous (combined datasets)	/	↑Abundance	Southwell et al. (2015)
		North‐East Antarctic Peninsula		1995–2023	Yearly	/	↓Abundance	Juáres et al. (2024)
		South Orkney Islands		1978–2016	Yearly	/	↓Abundance, ns Breeding success	Dunn et al. (2016)
				1978–2004	Yearly	↓SST, ↑Air temperature, ↓SIE	↓Abundance	Forcada et al. (2006)

				1979–1992	Yearly	ns SIE, ns SID	ns Abundance, ns Breeding success	Trathan et al. (1996)
				1995–2023	Yearly	/	↓Abundance	Juáres et al. (2024)
				1997–2001	Yearly	↓Abundance of prey (krill)	ns Abundance, ns Breeding success, ns Diet	Lynnes et al. (2004)
				1997–2006	Yearly	/	ns Diet	Rombolá et al. (2012)
		South Shetland Islands	128	1977–1996	Yearly	/	↓Abundance	Sierakowski et al. (2017)
				1981–2013	Yearly	/	↓Abundance, ns Breeding success, ns Survival	Hinke et al. (2017)
			128; 151	1990–1996	Yearly	/	↓Abundance	Ciaputa and Sierakowski (1999)
				1991–2009	Yearly	ns Air temperature	ns Breeding success, ns Phenology	Hinke et al. (2012)
			132	1995–2007	Yearly	/	↓Abundance, ↓Breeding success	Carlini et al. (2009)
				1995–2014	Yearly	/	↓Abundance, ↓Breeding success	Juáres et al. (2015)
				1995–2023	Yearly	/	↓Abundance	Juáres et al. (2024)
				2003–2015	Yearly	/	ns Diet	Juáres et al. (2018)
			128	2009–2012	Yearly	/	↓Abundance	Petry et al. (2016)
		Victoria Land		1965–1987	1965–1970, 1974–1987	/	↑Abundance	Wilson (1990)
				1975–2003	Yearly	ns SIE, ns SAT, ns SOI, ns Chlorophyll, ns Sedimentation	↓Abundance	Ducklow et al. (2007)
				1981–1987	Yearly	/	↑Abundance	Taylor et al. (1990)
				1981–2012	Yearly	/	ns Abundance	Lyver et al. (2014)
				1983–2010	Yearly	↑Air temperature, ↑Habitat availability	↑Abundance	LaRue et al. (2013)
				1995–2005	Yearly, excluding 2001 and 1999	/	ns Abundance, ns Breeding success	Pezzo et al. (2007)
			1997–2000	Yearly	/	↑Abundance, ns Breeding success, ns Growth, ↑Foraging distance	Ainley et al. (2004)

		West Antarctic Peninsula		1979–2007	Yearly	/	↓Abundance, ns Distribution	Lynch et al. (2012)
				1987–2016	Yearly	Local‐scale climate predictors of breeding success—↑SAM, ns SOI, ns ENSO, ↑Air temperature; ns Precipitation; ↑Wind; ns SID; ns SIR; ns SIE^	ns Breeding success ↓Abundance, ns Breeding season	Cimino et al. (2014); Cimino et al. (2019)
				1994–2000	Yearly		ns Abundance	Naveen et al. (2000)
				1995–2005	Yearly, excluding 1999/2000 and 2003/2004	/	↓Abundance, ns Breeding success	Carlini et al. (2007)
				2003–2008	Yearly	↑ Tourism	↓Abundance, ns Breeding success	Lynch et al. (2010)
				2009–2014	Yearly	/	ns Diet	Pickett et al. (2018)
		Wilkes Land		1959–1993	Yearly	↑Human visitation	↑Abundance, ns Breeding success, *Distribution	Woehler et al. (1994)
				1979–2010	Non‐continuous (combined datasets)	/	↑Abundance	Southwell and Emmerson (2015)
	Chinstrap Penguin—*Pygocelis antarcticus*	South Orkney Islands		1978–2016	Yearly	/	↓Abundance, ns Breeding success	Dunn et al. (2016).
				1978–2004	Yearly	↓SST, ↑Air temperature, ↓SIE	↓Abundance	Forcada et al. (2006)
				1979–1992	Yearly	ns SIE, ns SID	↓Abundance, ns Breeding success	Trathan et al. (1996)
				1997–2001	Yearly	↓Abundance of prey (krill)	ns Abundance, ns Breeding success, ns Diet	Lynnes et al. (2004)
				1997–2002	Yearly	/	ns Breeding success, ns Diet	Rombolá et al. (2006)
				1997–2006	Yearly	/	ns Diet	Rombolá et al. (2012)
		South Shetland Islands	128	1977–1996	Yearly	/	↓Abundance	Sierakowski et al. (2017)
				1985–2014	Yearly	/	↓Abundance	Petry et al. (2018)
			128; 151	1990–1996	Yearly	/	↓Abundance	Ciaputa and Sierakowski (1999)
				2002–2007	Yearly	/	ns Behavior, ns Diet	Miller and Trivelpiece (2008)
		128	2009–2012	Yearly	/	ns Abundance	Petry et al. (2016)

		West Antarctic Peninsula		1975–2003	Yearly	ns SIE, ns SAT, ns SOI, ns Chlorophyll, ns Sedimentation	↑Abundance	Ducklow et al. (2007)
				1979–2007	Yearly	/	↓Abundance, ns Distribution	Lynch et al. (2012)
				1994–2000	Yearly	/	ns Abundance	Naveen et al. (2000)
	Emperor Penguin— *Aptenodytes forsteri*	Adelie Land		1950–2004	Yearly	↑SAM, ↓SOI, ns Air temperature, ↓MSA, ns SIL	ns Breeding season	Barbraud and Weimerskirch (2006)
				1952–2000	Yearly	ns Air temperature, ns SST, ns SIE, ns SLP	↓Abundance, ns Breeding success	Barbraud and Weimerskirch (2001)
				1955–1999	Yearly	Impact of airstrip development	ns Abundance, ↓Breeding success	Micol and Jouventin (2001)
				1962–2009	Sporadically	Comparisons in population dynamics with Barbraud and Weimerskirch (2001)	↓Abundance, ns Breeding success	Barbraud et al. (2011)
				1963–2002	Yearly	ns SIC, ns SIE, ns Air temperature, ns SOI	↓Abundance, ns Breeding success	Jenouvrier et al. (2005)
				1971–1998	Yearly	↑SAM; ↑SIC	↑Juvenile survival	Abadi et al. (2017)
		Victoria Land		1957–2010	Yearly 1988–2010 compared with historic data 1957–1975	ns Air temperature; ns SIE	ns Abundance	Robertson et al. (2014)
				2000–2012	Yearly	/	ns Abundance	Kooyman and Ponganis (2017)
				1983–2005	Yearly	ns SIE, ns SST, ns SOI, ns SAM	ns Abundance	Barber‐Meyer et al. (2008)
		West Antarctic Peninsula		1950–2008	Yearly	↑Air temperature; ↓Fast ice duration	↓Abundance	Trathan et al. (2011)
	Gentoo Penguin—*Pygocelis papua*	South Orkney Islands		1978–2016	Yearly	/	↑Abundance, ns Breeding success	Dunn et al. (2016)
				1978–2004	Yearly	↓SST, ↑Air temperature, ↓SIE	↑Abundance	Forcada et al. (2006)
		South Shetland Islands	128	1977–1996	Yearly	/	↓Abundance	Sierakowski et al. (2017)
				1985–2014	Yearly	/	↓Abundance	Petry et al. (2018)
			128; 151	1990–1996	Yearly	/	↑↓Abundance	Ciaputa and Sierakowski (1999)
			1991–2009	Yearly	ns Air temperature	ns Breeding success, ns Phenology	Hinke et al. (2012)

			132	1995–2007	Yearly	/	↑Abundance, ↑Breeding success	Carlini et al. (2009)
				2002–2008	2002–2005, 2008	/	ns Behavior, ns Breeding success	Miller et al. (2009)
			128	2009–2012	Yearly	/	↓Abundance	Petry et al. (2016)
		West Antarctic Peninsula		1975–2003	Yearly	ns SIE, ns SAT, ns SOI, ns Chlorophyll, ns Sedimentation	↑Abundance	Ducklow et al. (2007)
				1979–2007	Yearly	/	↑Abundance, ↑Distribution	Lynch et al. (2012)
				1994–2000	Yearly	/	ns Abundance	Naveen et al. (2000)
				1996–2008	Yearly	↑Tourist visitation	↓Abundance	Trathan et al. (2008)
				1996–2016	Yearly	↑Tourist visitation; ↑SAT; ↓SIM	↓Abundance, ↓Breeding success	Dunn et al. (2019)
				2003–2008	Yearly	↑Tourism	↑Abundance, ns Breeding success	Lynch et al. (2010)
				2006–2018	2007, 2007, 2011, 2018	↑Moss bed destruction	*Behavior	Dykyy and Bedernichek (2022)
				2009–2014	Yearly	/	ns Diet	Pickett et al. (2018)
	King Penguin— *Aptenodytes patagonicus*	South Shetland Islands	128; 151	1977–2017	Yearly	/	↑Abundance	Gryz et al. (2019)
	Macaroni Penguin— *Eudyptes chrysolophus*	South Shetland Islands		1985–2014	Yearly	/	ns Abundance	Petry et al. (2018)
	West Antarctic Peninsula		1994–2000	Yearly	/	ns Abundance	Naveen et al. (2000)
**Marine Birds**	Antarctic Petrel— *Thalassoica antarctica*	Adelie Land		1950–2004	Yearly	↑SAM, ↓SOI, ns Air temperature, ↓MSA, ns SIL	*Breeding season	Barbraud and Weimerskirch (2006)
		East Antarctica		1984–1996	Sporadically	ns Air temperature, ns Wind, ns Precipitation, Impact of predation	ns Survival, ns Breeding success	Van Franeker et al. (2001)
	Antarctic Shag— *Leucocarbo bransfieldensis*	South Orkney Islands		1978–2021	Sporadically	/	↓Breeding pairs	Dunn et al. (2022)

		South Shetland Islands	128	1977–1996	Yearly	/	ns Abundance	Sierakowski et al. (2017)
				1985–2014	Yearly	/	ns Abundance	Petry et al. (2018)
				1988–2010	Sporadically	/	↓Abundance	Casaux and Barrera‐Oro (2016)
			128	2009–2012	Yearly	/	↓Abundance	Petry et al. (2016)
		West Antarctic Peninsula		1975–2018	Yearly	/	↑Abundance	Phillips et al. (2019)
				1994–2000	Yearly	/	↓Abundance	Naveen et al. (2000)
	Antarctic Tern— *Sterna vittata*	South Shetland Islands	128	1977–1996	Yearly	/	↓Abundance	Sierakowski et al. (2017)
				1985–2014	Yearly	/	ns Abundance	Petry et al. (2018)
			128	2009–2012	Yearly	/	↓Abundance	Petry et al. (2016)
	Black‐bellied Storm Petrel— *Fregetta tropica*	South Shetland Islands	128	1977–1996	Yearly	/	ns Abundance	Sierakowski et al. (2017)
	Brown Skua— *Stercorarius antarcticus*	South Shetland Islands	128	1977–1996	Yearly	/	ns Abundance	Sierakowski et al. (2017)
				1985–2014	Yearly	/	ns Abundance	Petry et al. (2018)
				2002–2015	Yearly	/	ns Abundance, ↓Breeding success	Krietsch et al. (2016)
			128	2009–2012	Yearly	/	ns Abundance	Petry et al. (2016)
				2020–2024	Yearly	/	ns Abundance, ns Breeding success	Komarowska et al. (2025)
	Cape Petrel—*Daption capanse*	Adelie Land		1950–2004	Yearly	↑SAM, ↓SOI, ns Air temperature, ↓MSA, ns SIL	*Breeding season	Barbraud and Weimerskirch (2006)
				1984–1999	Yearly	Impact of airstrip development	ns Abundance, ns Breeding success	Micol and Jouventin (2001)
		South Shetland Islands	128	1977–1996	Yearly	/	ns Abundance	Sierakowski et al. (2017)
				1985–2014	Yearly	/	↓Abundance	Petry et al. (2018)
				2001–2020	Yearly, excluding 2002 and 2003	/	↓Abundance	Braun et al. (2021)
			128	2009–2012	Yearly	/	↓Abundance	Petry et al. (2016)
Kelp gull— *Larus dominicanus*	South Shetland Islands	128	1977–1996	Yearly	/	ns Abundance	Sierakowski et al. (2017)

				1985–2014	Yearly	/	ns Abundance	Petry et al. (2018)
			128	2009–2012	Yearly	/	↓Abundance	Petry et al. (2016)
	Pale‐faced Sheathbill— *Chionis albus*	South Shetland Islands	128	1977–1996	Yearly	/	ns Abundance	Sierakowski et al. (2017)
	Snow Petrel— *Pagodroma nivea*	Adelie Land		1950–2004	Yearly	↑SAM, ↓SOI, ns Air temperature, ↓MSA, ns SIL	*Breeding season	Barbraud and Weimerskirch (2006)
				1984–1999	Yearly	Impact of airstrip development	ns Abundance, ns Breeding success	Micol and Jouventin (2001)
		West Antarctic Peninsula		1962–2001	Yearly	ns SIC, ns SIE, ns Air temperature, ns SOI	ns Abundance, ns Breeding success	Jenouvrier et al. (2005)
		Wilkes Land		1984–2003	Yearly	ns SIE, ns SST	ns Breeding success	Olivier et al. (2005)
	South Polar Skua— *Stercorarius maccormicki*	Adelie Land		1950–2004	Yearly	↑SAM, ↓SOI, ns Air temperature, ↓MSA, ns SIL	*Breeding season	Barbraud and Weimerskirch (2006)
				1964–1997	Yearly	Impact of airstrip development	↑Abundance, ns Breeding success	Micol and Jouventin (2001)
				1968–2014	Yearly	ns Air temperature; ns SIC; ns SST	↑Abundance	Pacoureau et al. (2018)
		South Shetland Islands	128	1977–1996	Yearly	/	ns Abundance	Sierakowski et al. (2017)
				2002–2015	Yearly	/	↓Abundance, ↓Breeding success	Krietsch et al. (2016)
			128	2009–2012	Yearly	/	ns Abundance	Petry et al. (2016)
				2020–2024	Yearly	/	ns Abundance, ns Breeding success	Komarowska et al. (2025)
		West Antarctic Peninsula		1975–2018	Yearly	/	↑Abundance	Phillips et al. (2019)
	Southern Giant Petrel— *Macronectes giganteus*	Adelie Land		1950–1999	Yearly	Impact of airstrip development	ns Abundance, ↓Breeding success	Micol and Jouventin (2001)
				1950–2004	Yearly	↑SAM, ↓SOI, ns Air temperature, ↓MSA, ns SIL	ns Breeding season	Barbraud and Weimerskirch (2006)
		South Shetland Islands	128	1977–1996	Yearly	/	ns Abundance	Sierakowski et al. (2017)
				1985–2014	Yearly	/	↑Abundance	Petry et al. (2018)
			2009–2012	Yearly	/	ns Abundance	Petry et al. (2016)

		West Antarctic Peninsula		1994–2000	Yearly	/	ns Abundance	Naveen et al. (2000)
	Southern Fulmar— *Fulmarus glacialoides*	Adelie Land		1950–2004	Yearly	↑SAM, ↓SOI, ns Air temperature, ↓MSA, ns SIL	*Breeding season	Barbraud and Weimerskirch (2006)
				1962–1999	Yearly	Impact of airstrip development	ns Abundance, ↑Breeding success	Micol and Jouventin (2001)
	Wilson's Storm Petrel— *Oceanites oceanicus*	Adelie Land		1950–2004	Yearly	↑SAM, ↓SOI, ns Air temperature, ↓MSA, ns SIL	ns Breeding season	Barbraud and Weimerskirch (2006)
		South Shetland Islands	128	1977–1996	Yearly	/	ns Abundance	Sierakowski et al. (2017)
			1978–2020	Yearly	Population decline linked to interannual variation in SIE, SST, SAM, Air temperature, Precipitation, Wind speed^	↓Abundance, ↓Breeding success, ns Growth	Ausems et al. (2023)
**Marine Mammals**	Antarctic Fur Seal— *Arctocephalus gazella*	South Shetland Islands		1958–2002	20 breeding seasons	/	↑Abundance	Hucke‐Gaete et al. (2004)
				1986–1995	Yearly	↑Predation, ns Human disturbance	↑↓Abundance	Boveng et al. (1998)
			128	1988–1995	Yearly	/	ns Abundance	Salwicka and Rakusa‐Suszczewski (2002)
			128	1993–1997	Yearly	/	ns Diet	Ciaputa and Siciński (2006)
				1996–1998	Yearly	/	*Diet	Daneri et al. (2008)
		South Orkney Islands		1996–2002	Yearly	/	ns Abundance, ns Breeding success, ns Survival	Hofmeyr et al. (2005)
				1977–2008	Yearly	ns Fast ice	ns Abundance	Waluda et al. (2010)
	Crabeater Seal— *Lobodon carcinophagus*	McMurdo Sound; West Antarctic Peninsula		1970–1987	Yearly	/	ns Abundance, ns Breeding success	Testa et al. (1991)
		South Shetland Islands	128	1988–1995	Yearly	/	↓Abundance	Salwicka and Rakusa‐Suszczewski (2002)
		West Antarctic Peninsula		2001–2007	2001, 2002 and 2007	/	ns Diet	Hückstädt et al. (2012)

	Elephant Seal— *Mirounga leonina*	South Shetland Islands	128	1988–1995	Yearly	/	ns Abundance	Salwicka and Rakusa‐Suszczewski (2002)
	Leopard Seal— *Hydrurga leptonyx*	South Shetland Islands		1987–1995	Yearly	/	ns Behavior	Hiruki et al. (1999)
			128	1988–1995	Yearly	/	ns Abundance	Salwicka and Rakusa‐Suszczewski (2002)
	Weddell Seal— *Leptonychotes weddellii*	East Antarctica	127	1958–2016	1958, 1961, 1999, 2000, 2006, 2010, 2011, 2012, 2016	/	ns Abundance	Golubev (2023)
		McMurdo Sound		1970–1987	Yearly	/	ns Abundance, ns Breeding success	Testa et al. (1991)
		Princess Elizabeth Land		1973–2000	Yearly, excluding 1997	/	ns Abundance, ns Breeding success, ns Survival	Lake et al. (2008)
		South Shetland Islands	128	1988–1995	Yearly	/	↓Abundance	Salwicka and Rakusa‐Suszczewski (2002)
		Victoria Land		1959–2012	1959–1968, 2008–2012	ns SIE	↓Abundance	Ainley et al. (2015)
				1963–2000	Yearly	/	ns Abundance, ns Survival	Cameron and Siniff (2004)
				1969–2010	Yearly	/	ns Abundance, ns Breeding success, ns Survival	Chambert et al. (2012)
				1973–1989	Yearly	/	ns Survival	Hastings et al. (1999)
				1989–1993	Yearly	/	ns Diet	Burns et al. (1998)
				2010–2012	Yearly	/	ns Diet	Goetz et al. (2017)
		West Antarctic Peninsula		1970–1987	Yearly	/	ns Abundance, ns Breeding success	Testa et al. (1991)
			132	2003–2005	Yearly	/	*Diet	Daneri et al. (2018)
Ross Seal— *Ommatophoca rossii*	East Antarctica	127	1915–2015	1915, 1958, 2007, 2009, 2020, 2012, 2015	/	ns Abundance	Golubev (2023)

*Note:* Note that some studies predate ASPA designation.

Abbreviations: * = significant change, / = no measurement, ^ = short‐term changes in environmental parameters which resulted in long‐term changes in biological parameters, + = studies that may overlap results within corresponding monitoring programs, ↑ = significant increase, ↓ = significant decrease, ENSO = El Niño‐Southern Oscillation, MSA = methane sulfuric acid, ns = non‐significant result, SAM = Southern Annular Mode, SIC = sea ice concentration, SID = sea ice drift, SIE = sea ice extent, SIL = sea ice lows, SIR = sea ice retreat, SOI = Southern Ocean Index, SST = sea surface temperature.

**TABLE 3:** Long‐term monitoring studies of nearshore marine organisms in Antarctic Maritime and Continental regions.

Functional group	Species/taxa	Location	Declared ASPA	Study duration	Study years	Environmental parameters	Results/biological response	References
**Fish**	*Gobionotothen gibberifrons*	South Shetland Islands		1983–1999	Yearly	Recovery of key species targeted by fisheries	↓Abundance	Barrera‐Oro et al. (2000)+
				1983–2010	Yearly	Recovery of key species targeted by fisheries	↓Relative abundance, ↑Growth	Marschoff et al. (2012)+
				2011–2016	Yearly	Recovery of key species targeted by fisheries	ns Abundance	Barrera‐Oro et al. (2017)+
	*Notothenia corriopes*	South Shetland Islands		1983–1999	Yearly	Recovery of key species targeted by fisheries	ns Abundance	Barrera‐Oro et al. (2000)+
				1983–2010	Yearly	Recovery of key species targeted by fisheries	↓Abundance, ns Growth	Marschoff et al. (2012)+
				2011–2016	Yearly	Recovery of key species targeted by fisheries	ns Abundance	Barrera‐Oro et al. (2017)+
	*Notothenia rossii*	South Shetland Islands		1983–1999	Yearly	Recovery of key species targeted by fisheries	↓Abundance	Barrera‐Oro et al. (2000)+
				1983–2010	Yearly	Recovery of key species targeted by fisheries	↑Relative abundance, ↓Growth	Marschoff et al. (2012)+
				2011–2016	Yearly	Recovery of key species targeted by fisheries	↑Abundance	Barrera‐Oro et al. (2017)+
	*Pagothenia* spp.	McMurdo Sound		2005–2011	2005, 2010, 2011	↑Fisheries pressure on key predator *Dissostichus mawsoni*	↑Biodiversity	Buckley (2013)
	*Trematomus* spp.	McMurdo Sound		2005–2011	2005, 2010, 2011	↑Fisheries pressure on key predator *Dissostichus mawsoni*	↑Biodiversity	Buckley (2013)
	*Nototheniidae* spp.	West Antarctic Peninsula		2006–2017	2006–2008, 2010, 2017	/	↑Relative abundance, *Species composition	Trokhymets et al. (2022)
	*Bathydraconiae* spp.	West Antarctic Peninsula		2006–2017	2006–2008, 2010, 2017	/	ns Relative abundance, ns Species composition	Trokhymets et al. (2022)
	*Channichthyidae* spp.	West Antarctic Peninsula		2006–2017	2006–2008, 2010, 2017	/	↑Relative abundance, ns Species composition	Trokhymets et al. (2022)
	*Harpagiferidae* spp.	West Antarctic Peninsula		2006–2017	2006–2008, 2010, 2017	/	ns Relative abundance, ns Species composition	Trokhymets et al. (2022)
**Marine Benthos**	Bryozoan	West Antarctic Peninsula		1997–2013	1997, 2001–2003, 2006–2007 and 2009, 2011, 2013	↑Ice scour	↓Abundance, ↓Competition	Barnes et al. (2014)+
				1997–2009	1997, 2001–2003, 2006–2007 and 2009	↑Ice scour	↓Survival	Barnes and Souster (2011)+
				2009–2014	Yearly	Response to ice scour disturbance	ns Abundance	Robinson, Barnes, and Morley (2020)
	Encrusting Fauna	South Shetland Islands		1994–2010	1994, 1998, 2000	Glacial retreat	↓↑Abundance, ↓Biodiversity	Sahade et al. (2015)
				2016–2018	Yearly	/	↓Biodiversity	Angulo‐Preckler et al. (2023)
		West Antarctic Peninsula		1997–2015	Yearly	ns SST, ns Salinity, ns PAR, ns Ice scour	ns Survival, ns Growth, ns Carbon accumulation	Barnes (2017)
	Infauna	McMurdo Sound		1988–1998	Yearly	Response of benthic community to sewerage outfall contamination	↓Abundance, ↓Biodiversity	Conlan et al. (2004)+
				1988–2004	Yearly	Response to sewerage outfall treatment	↑Abundance, ns Biodiversity	Conlan et al. (2010)+
				2000–2004	2000, 2003, 2004	Response to contamination gradient	ns Community composition, ns Biomass, ns Abundance	Morehead et al. (2008)
		Victoria Land		2000–2013	2000 to 2013, except for 2001 and 2002	Response of benthic community to contamination	ns Abundance, ns Biodiversity, ns Growth	Palmer et al. (2021)
	Macrofauna spp.	Victoria Land		2001–2009	Yearly, except for 2004 and 2007	Response to changing iceberg dynamics	↑Abundance, ns Biodiversity	Thrush and Cummings (2011)
	Megafauna spp.	Victoria Land		2001–2009	Yearly, except for 2004 and 2007	Response to changing iceberg dynamics	↑Abundance, ns Biodiversity	Thrush and Cummings (2011)
	Microphytobenthos	South Shetland Islands		1996–2005	1996/1997–1997/1998, 2004/2005	*Sediment properties	*Physiology	de Skowronski et al. (2009)
	Nemertean spp.	West Antarctic Peninsula		1997–2000	Yearly	ns SIC, ns SIE, ns Air temperature	ns Reproductive development	Grange et al. (2011)
**Marine Vegetation**	Algae spp.	South Shetland Islands		2010–2013	Yearly	/	ns Abundance, ns Biodiversity	Pellizzari et al. (2017)
		Victoria Land		1989–2004	1989/90, 1993–1995, 1997/98, 1999–2001, 2002–2004.	/	ns Abundance, ns Biodiversity	Majewska et al. (2013)
	Seaweed	South Shetland Islands		2007–2013	2007, 2008, 2009 and 2013	↓Annual ice foot cover, ↓Snow days	↑Abundance, ns Biodiversity	Marcías et al. (2017)
**Plankton**	Phytoplankton	South Shetland Islands		1991–2009	Yearly	↑Summer SST; ns Salinity; ns Total particulate matter; SIC; ↑Air temperature; *Wind speed	ns Abundance	Schloss et al. (2012)
				1996–2008	Yearly, excluding 1997	/	ns Abundance, ns Biodiversity	Lee et al. (2015)
				2010–2020	Yearly, excluding 2015	↑SST, ns Salinity, ns Air temperature, ns Turbidity, ns Upper mixed depth, ns Inorganic material	↑Abundance, ↑Biodiversity, ↑Biomass	Antoni et al. (2024)
		West Antarctic Peninsula		1991–1994	Yearly	ns Air temperature, ns Water temperature, ns Snow cover, ns Wind speed & Direction	ns Species composition, ns Biomass, ns Primary productivity	Moline and Prézelin (1996)+
				1991–2000	Yearly	ns SIE	ns Biomass, ns Primary Productivity	Smith et al. (2001)+
				1997–2010	Yearly	↑SIR, ↑SST	↑Primary productivity	Moreau et al. (2015)
	Plankton	South Shetland Islands		2010–2020	Yearly, excluding 2015	↑SST, ns Salinity, ns Air temperature, ns Turbidity, ns Upper mixed depth, ns Inorganic material	↑Abundance, ↑Biodiversity, ↑Biomass	Antoni et al. (2024)
		Vestfold Hills		1978–1987	1978, 1983–1987	/	*Biodiversity	Eslake et al. (1991)
		West Antarctic Peninsula		1997–2015	Yearly	ns SST, ns Salinity, ns PAR, ns Ice scour	ns Survival, ns Growth, ns Carbon accumulation	Barnes (2017)
				2017–2020	2017/18, 2018/19, 2020	↑SIR, ns SST, ns Tides	↑Biomass (zooplankton), ns Phytoplankton Biomass, ns Size structure, *Species composition, *Abundance	Conroy et al. (2023)+

*Note:* Note that some studies predate ASPA designation.

Abbreviations: * = significant change, / = no measurement, + = studies that may overlap results within corresponding monitoring programs, ↑ = significant increase, ↓ = significant decrease, ns = non‐significant result, SIC = sea ice concentration, SID = sea ice drift, SIE = sea ice extent, SIL = sea ice lows, SIR = sea ice retreat, SST = sea surface temperature.

### 3.4 Spatial Distribution

**FIGURE 3:**
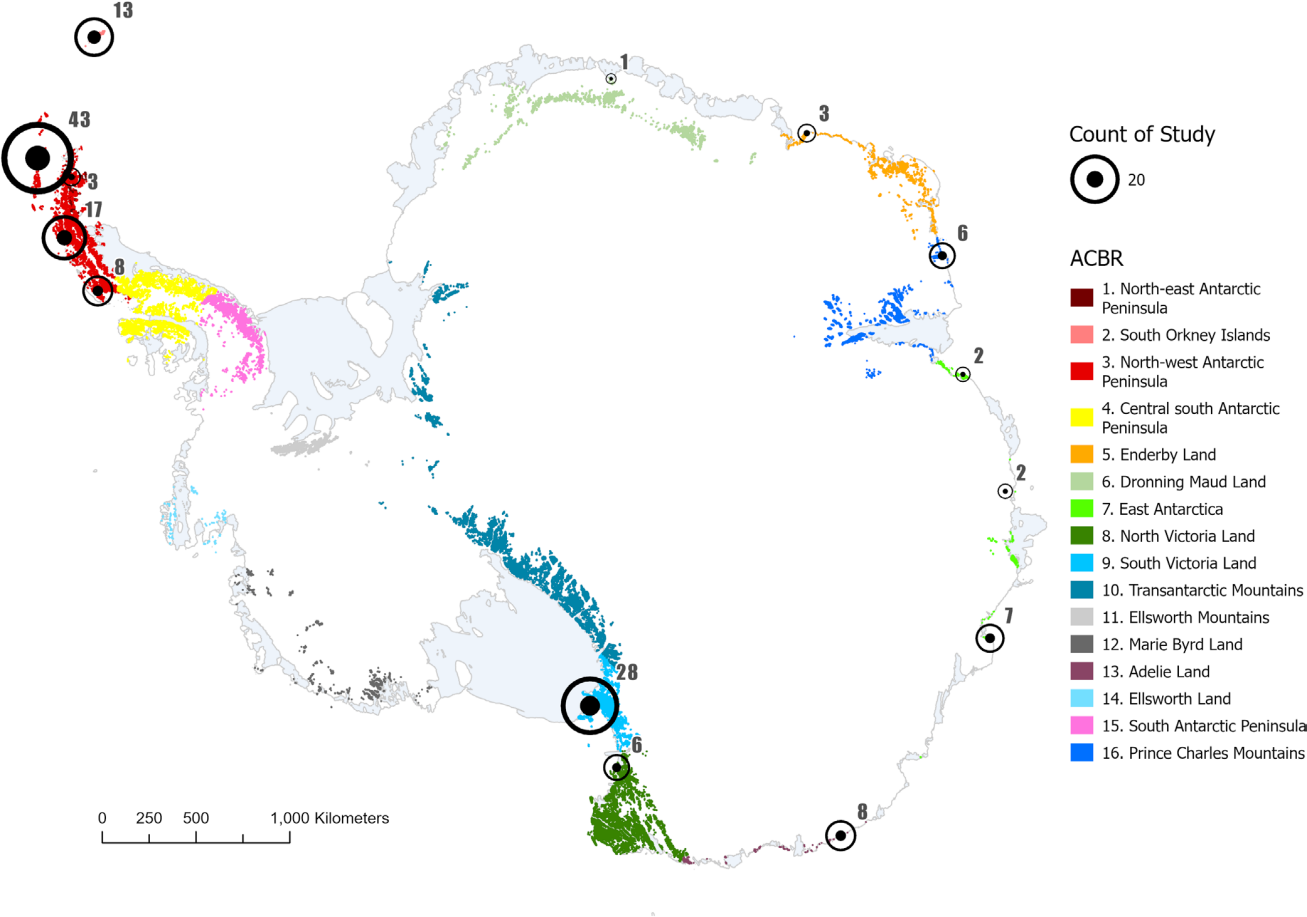
Count of long‐term nearshore or terrestrial monitoring studies across Antarctica. Dot size indicates the number of studies at a given location within the 16 Antarctic Conservation Biogeographic Regions (ACBR) (Terauds et al. 2012; Terauds and Lee 2016). Studies involving sites from multiple ACBRs are included once in each ACBR. Where multiple papers have been published about a single site, all are included.

Spatial bias was widely evident across LTM locations in Antarctica. Sixty percent of the 136 publications were focused on sites in Maritime Antarctica, including along the Antarctic Peninsula (Figure 3). Comparatively, monitoring across Continental Antarctica was reported in less than 40% of total studies. This difference is unsurprising given the varied accessibility and necessary resources to support research efforts across the continent. Although a higher proportion of LTM has been undertaken in the Maritime Antarctic region, where areas are richer in biodiversity and abundance (Convey et al. 2014; Walshaw et al. 2024), there are continental sites of high biological importance that are lacking similar efforts, e.g., ACBRs 5—*Enderby Land*, 6—*Dronning Maud Land*, 7—*East Antarctica*, and 16—*Prince Charles Mountains* (Patterson et al. 2024; Toth et al. 2025; Walshaw et al. 2024). Considering its vast size and vital role in global climate, the general lack of LTM in Antarctica remains striking.

It is likely the uneven spatial distribution of LTM studies found reflects a degree of sampling bias. Monitoring was most common on species or communities near research stations, with two‐thirds of monitoring sites occurring within 20 km of permanent stations: 60% of these sites occurring within a more conservative 5‐km station footprint (supporting information and Figure S1). Over half of the approximately 30 LTM sites situated further than 50 km from a permanent research facility were nearshore marine‐based studies. Similarly, Patterson et al. (2024) identified strong sampling bias of biological specimen records from Antarctica to be close to research stations, but in only half of the ACBRs. Across global monitoring programs, proximity to human development has been identified as a common spatial bias of ecological research (Trimble and van Aarde 2012). This is exacerbated when conducting research in remote and challenging locations, such as Antarctica. Many research stations are located on the limited ice‐free land of Antarctica's coastline (Brooks, Jabour, et al. 2019), close to terrestrial biota (Brooks et al. 2024; Lee et al. 2017; Lee, Waterman, et al. 2022). Researchers and National Antarctic Programs are probably more likely to establish LTM sites close to stations where they are more easily funded and can be measured more frequently than remote sites, which are more difficult and costly to reach. However, as the human footprint in Antarctica continues to grow, it is vital that the biota in the surrounding areas of research station infrastructure are monitored to assess the impact of human activities on Antarctic biological systems.

One might also expect much of the monitoring to occur within ASPAs as part of the ASPA management review process. However, only 10% of the published studies were undertaken in ASPAs (Tables 1, 2, 3). Shaw et al. (2014) previously demonstrated the limitations of current specially protected area coverage, which, considering this review, shows many communities are both under‐monitored and under‐protected across Antarctica.

For five of the 16 ACBRs, no long‐term biological monitoring of terrestrial biological communities was found. These are ACBRs 10—*Transantarctic Mountains*, 11—*Ellsworth Mountains*, 12—*Marie Byrd Land*, 14—*Ellsworth Land*, and 15—*South Antarctic Peninsula* (Figure 3). These ecoregions have low vertebrate diversity but do have unique microbial, invertebrate, and plant diversity, which currently remains unmonitored. These lesser disturbed and more remote ecoregions of Antarctica remain under‐sampled, under‐represented, and under‐monitored, coinciding with other literature, databases, and surveys (Figure 3; Borgmeier et al. 2025; Liu et al. 2022; Patterson et al. 2024; Pertierra et al. 2025; Terauds et al. 2025; Toth et al. 2025). Understanding how these remote communities are responding to change remains an important research question.

These spatial biases in Antarctic LTM are not always the result of researcher preference or funding limitations. Many of the gaps and disparities are the result of the harsh environment, which makes research, particularly large‐scale, long‐term data collection, difficult and logistically challenging, along with differences in ease of accessibility across the continent. Ultimately, however, this still results in under‐representation of certain communities and genera.

Combining ground, airborne, and satellite measurements can provide invaluable datasets for monitoring nearshore and terrestrial communities (Colesie et al. 2022; Fretwell et al. 2023; Piazza et al. 2020; Walshaw et al. 2024). Development and application of remote sampling methods and techniques, particularly when coupled with field data for ground‐truthing, will aid in monitoring of hard‐to‐access regions across Antarctica (Malenovský et al. 2017; Turner et al. 2024). Satellite data is increasingly used (Colesie et al. 2025; Fretwell et al. 2023; Walshaw et al. 2024) particularly in nearshore coastal regions, and over the past 15 years, remotely piloted systems have also been deployed, but so far only a few have been used for LTM (Colesie et al. 2022; Jansen et al. 2018; Turner et al. 2024). Additionally, there is potential to couple shallow‐water moorings, which are commonly used to collect physical and biogeochemical data, to monitor nearshore ecosystems, e.g., settlement of scallops as indicators of water temperature and flow direction in the Ross Sea (Schiaparelli and Aliani 2019).

### 3.5 Taxonomic Focus

**FIGURE 4:**
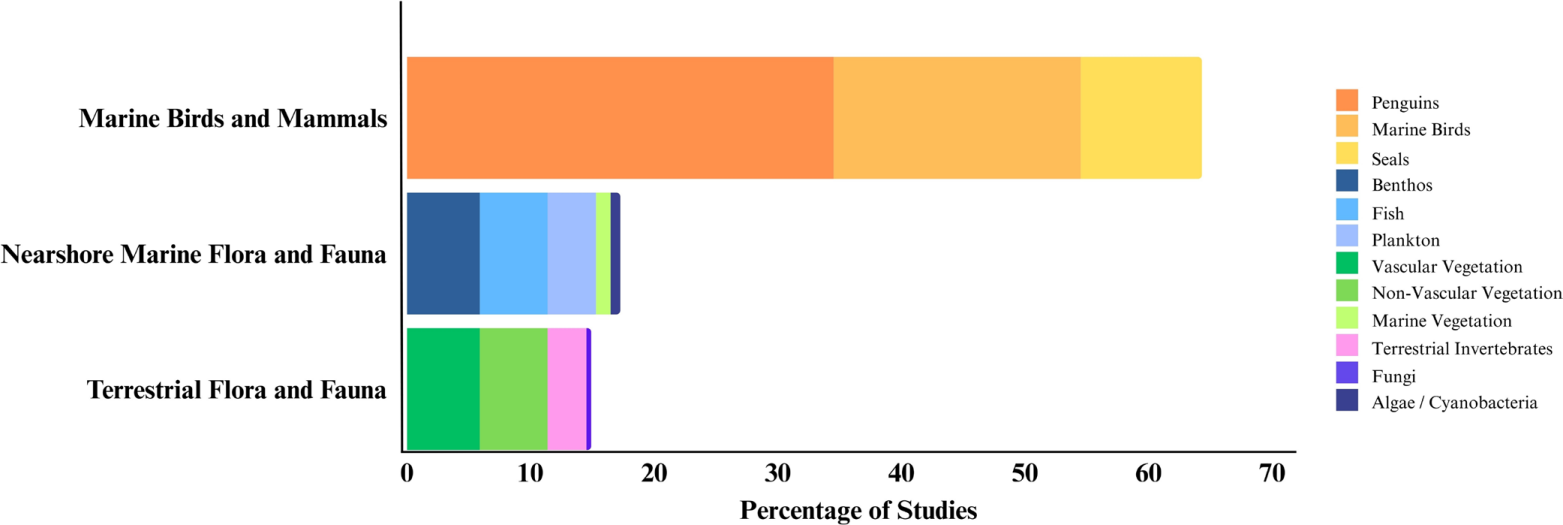
Percentage of studies monitoring Antarctic biota by species type within groups: Marine birds and mammals; nearshore marine flora and fauna; and terrestrial flora and fauna including vegetation, invertebrates, cyanobacteria, and fungi. Studies that monitored several species were included more than once.

Unsurprisingly, Antarctic long‐term biological monitoring shows a strong bias towards charismatic fauna (Figure 4; Tables 1, 2, 3). Most studies concentrated on marine birds and mammals (~65%), followed by other nearshore marine flora and fauna (~17%) (Figures 2 and 4). Conversely, 15% of publications described terrestrial groups, such as mosses, lichens, fungi, invertebrates, and microbes. Even within the biota groups, further bias is apparent; for example, monitoring of colony‐building species such as Adélie penguins and Weddell seals vastly outnumber those of solitary species, such as leopard seals (Table 2). This finding is in line with patterns observed in global LTM studies and ecological research in general (Bonnet et al. 2002).

Increased interest in charismatic fauna by both public and government bodies is likely to blame for this global bias (Mammola et al. 2020), meaning increased funding opportunities and public outreach can be achieved when a charismatic study species is adopted. Additionally, monitoring higher‐order marine predators, such as penguins or seals, can reflect broad dynamics in food webs and the marine environment (Hazen et al. 2019). Charismatic species can improve awareness of the impact of changing climates as well as the importance of conservation and biodiversity for the public. This can indirectly provide valuable opportunities for the conservation and monitoring of the more‐neglected species within similar habitats. Nevertheless, there is growing evidence that numerous Antarctic biological groups are sensitive to changing climates (Chown et al. 2022; Convey and Peck 2019). Therefore, they should also be considered important indicators of the health of the Antarctic ecosystem independent of their varying level of perceived value and interest. Considering the unique challenges facing Antarctic marine organisms, the low number of monitoring records in these sensitive communities is concerning. From the existing studies, it is apparent changes can be rapid (Dayton et al. 2016; Dunn et al. 2025; Thrush and Cummings 2011; Wienecke et al. 2024), and in many instances, there is a lack of adequate baseline data to compare to any future changes.

The lesser monitored communities and organisms of the Antarctic play vital roles in ecosystem function, the potential complexity of which is only recently being understood (Cary et al. 2010; Griffiths and Waller 2016). For the terrestrial and nearshore marine communities, which are highly sensitive to regional regime shifts, this lack of monitoring means we do not understand how entire communities are responding to the rapidly changing environment around them. What we do know is communities are already displaying shifts in composition, and community responses can be highly regional (Amsler et al. 2023; Bergstrom et al. 2021; Cannone et al. 2022; Robinson et al. 2018) demonstrating the need for continent‐wide monitoring across the diverse range of ecological communities in Antarctica.

### 3.6 Biological Parameters

Most studies reported significant shifts in measured biological parameters (Tables 1, 2, 3). As expected, the direction or nature of the reported results was highly dependent on not only study species but also on study location, highlighting the variability of responses across the continent. While some studies have found seemingly “positive” findings such as increases in abundance, biodiversity, productivity, etc., over time, these results detail large‐scale ecological shifts in response to warmer, wetter conditions (Cannone et al. 2022; Prather et al. 2019), from which we might expect species exclusions and extinctions to follow (e.g., Losapio et al. 2025; Roland et al. 2024 together with Colesie et al. 2025; Sancho et al. 2017).

**FIGURE 5:**
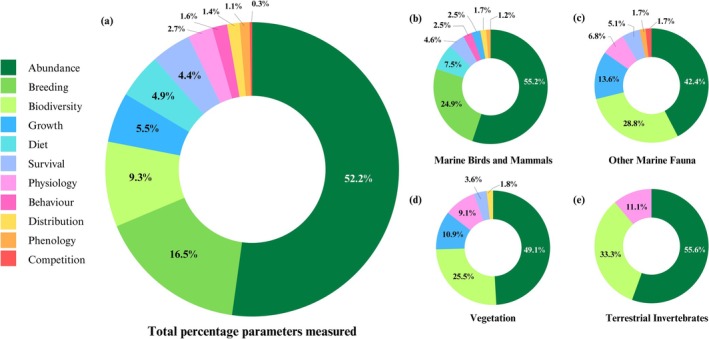
Percentage of parameters measured across all Antarctic biota (a) and within biota groups: Marine birds and mammals (b), other marine fauna (c), vegetation (d), and terrestrial invertebrates (e). Studies that monitored multiple biological parameters were included more than once.

Abundance or population size was the most frequently measured parameter across all studies (52%; Figure 5a), and within nearshore marine and terrestrial taxonomic groups (a range of 42%–56%; Figure 5b–e). Biodiversity was a key parameter across all biota groups (apart from marine birds and mammals, which were all single species studies), measured in 25%–33% of studies (Figure 5). Growth was another common biological characteristic that was monitored for most biota groups and was measured in 3%–14% of studies. Interestingly, survival was not monitored for terrestrial invertebrates (Figure 5e).

Whilst abundance is an important measure for population status and growth, most studies lacked parameters to determine population or community health. Parameters that account for health changes, such as growth rates, physiology, and phenology, are essential in understanding how species and communities may be responding to current and changing environmental pressures. Yet, less than 10% of studies considered these factors overall. Further taxonomic biases can be seen in the measured parameters as well, with plant groups and terrestrial invertebrate groups having far fewer parameters measured in comparison to marine birds and marine fauna. Although interestingly, physiological measures were more common for vegetation and invertebrates than marine fauna, and further, there were zero physiological LTM studies for marine birds and mammals. Discrepancies in the biological parameters measured may be due to the varied resources and expertise required for certain analyses, such as for DNA and physiological measurements, as well as the difficulties associated with collecting data or samples in remote locations.

### 3.7 Environmental Co‐Variates

While biological measures were the focus of this review, the use of environmental data to inform biological trends was evident in almost 60% of studies (Figure 2, Table 4). The impact of changing water availability and increased human disturbance were found to be key drivers of change across Antarctic biota. Of the studies which found significant changes in water availability over time, including deglaciation, changes in precipitation, snow melt, and snow cover, biotic responses were found to also shift significantly (e.g., Table 1). Similarly, studies which measured various responses of biota to increased human visitation and disturbance, such as construction and contamination, all found significant changes across biota. These changes were mostly negative, including reduced abundance, biodiversity, and breeding success (Tables 1, 2, 3); however, Micol and Jouventin (2001) highlight the vast disparities across species responses.

**TABLE 4:** Various environmental variables were reported alongside biological characteristics in Antarctic long‐term monitoring studies, here described as number and percentage of publications.

Environmental variable	Count	Percentage
Air temperature	30	22%
Sea ice characteristics	30	22%
Sea surface temperature	14	10%
Disturbance	14	10%
Precipitation/water availability	11	8%
Wind speed/direction	10	7%
Southern Oscillation Index	10	7%
Fisheries	10	7%
Southern Annular Mode	8	6%
Predation	6	4%
Glacial retreat/deglaciation	5	4%
Pollution	5	4%
Sediment properties	4	3%
El Niño–Southern Oscillation	3	3%
Salinity	3	2%
Solar visible radiation	3	2%
Conductivity	2	1%
Soil/moss temperature	2	1%
pH	1	1%

*Note:* Approximately 58% of studies analyzed environmental variables (see Tables 1, 2, 3). Studies that analyzed multiple environmental variables were counted more than once.

Of the two studies reported, the monitoring of invasive species on the continent increased propagule pressure originating from research bases (Newman et al. 2018), and newly modified Antarctic environments in response to changing climates, leaves these spaces vulnerable to invasion (Table 1). One of the only known invasive vascular plants, *P. annua* , was shown to increase in abundance in response to more favorable conditions following glacial retreat on King George Island (Olech 2010; Table 1).

The most reported environmental characteristics were air and sea surface temperature, along with sea ice characteristics and various forms of physical disturbance (Figure 2, Table 4). Sea ice properties together with air temperatures were common in LTM studies on marine birds and mammals (Table 2). Several environmental datasets were obtained from large‐scale meteorological observations and were not directly measured in the field. Few studies measured microclimate variables such as salinity, solar visible radiation, land surface temperature, and pH (Table 4). However, 51 studies did not compare or use environmental parameters to explain the reported trends in biological characteristics. This neglect could be due to a lack of climate data at the relevant scales needed to understand the biological responses recorded, including in situ measurements or validated satellite and climate records at fine resolution.

### 3.8 Method Inconsistency

The monitoring methods employed across the published studies were often highly variable. While the lesser monitored organisms clearly had limited room for variation owing to the low study numbers, variation between studies on marine birds and mammals was high. One major discrepancy between studies was sampling effort, i.e., the time allocated per survey and number of people sampling per study. When considering variations in abundance and biodiversity data, both within and between studies, sampling effort can change the precision of observations considerably (Ashcroft et al. 2017). Discrepancies in sampling effort can lead to erroneous conclusions about populations, where greater effort surveys will capture more individuals and species as well as estimate demographic parameters more accurately than lower effort surveys (Elphick 2008; Symons et al. 2018). When factors such as the number of counters, transect length, and quadrat numbers, search times, and total survey area vary among studies, sampling effort is affected and confidence in the inferences drawn from the data should be reduced. While some studies noted the number of observers (Carlini et al. 2009; Juáres et al. 2015; Petry et al. 2018), most others did not, leaving ambiguity in methodologies and also introducing potential variability and bias into the data. Further, ground counts, aerial counts, or satellite counts of bird and mammal populations all allow for varied degrees of accuracy and precision in population estimates (see references within Tables 1, 2, 3 for examples), while this is permissible for comparisons within a study, reliable comparisons between studies, locations, and organisms are then reduced. Other variations included differences in sampling type for vegetation studies, i.e., counts versus transects (Fowbert & Lewis Smith 1994; Park et al. 2012) or soil sampling depths for soil invertebrate studies (Andriuzzi et al. 2018; Sohlenius and Boström 2005).

Existing programs, such as CCAMLR, highlight some of the advantages of having strongly streamlined protocols. Several studies focusing on marine birds, mammals, and penguins referenced CCAMLR's standardized methods (CCAMLR 2004), which show promise in the uptake and utilization of standardized methods. Alternative methods, however, were commonly adopted by investigators, suggesting varying levels of compliance to prescribed protocols.

## 4 Recommendations

### 4.1 Better Approaches to Antarctic LTM

Considering the high level of bias and limitations in monitoring biota across Antarctic nearshore marine and terrestrial zones, there is a need for better approaches to monitoring, including uniform data collection, data sharing, and international collaborations in research efforts. The development of Antarctic‐wide observation systems, as proposed by the Antarctic Nearshore and Terrestrial Observation System (ANTOS, https://www.scar.org/science/antos/home/) and Antarctica InSync (https://www.antarctica‐insync.org/), aims to close the gaps in current monitoring by harmonizing data collection protocols and facilitating data sharing and collaborations.

Generally, published LTM practices across Antarctica have been developed with a specific scientific question posed by the primary investigators. As a consequence, monitoring studies are unharmonized and unevenly distributed across the continent. Therefore, we recommend that Antarctic biodiversity monitoring would benefit from more consistent and coordinated methods if it is to become an asset to conservation management, with the purpose of protecting Antarctica's biodiversity.

LTM is often difficult under typical 1‐ to 5‐year grant time frames, especially so in Antarctic science where it is compounded by the need for repeat access to the same remote field sites. Obtaining funding and logistical support, grant cycle after grant cycle, may be difficult and ultimately less attractive to researchers. Monitoring of environmental and biological data over the long term (decades) can be costly and therefore requires commitments and logistic support from a combination of National Antarctic programs, philanthropic and other funding agencies. It would be ideal to establish long‐term sites that have baseline data, synchronize existing infrastructure, e.g., by adapting already built and maintained weather stations that are in potential sentinel sites, and standardize protocols utilized by ongoing monitoring programs. There are limited examples of centrally organized and funded site‐based monitoring networks across the globe. Four such networks are the Integrated Carbon Observation System (ICOS), the Long‐Term Ecological Research (LTER) project, the National Ecological Observatory Network (NEON), and the Terrestrial Ecosystem Research Network (TERN). Since their inception, these programs have separately facilitated the publication of 1500+ journal articles, emphasizing the significance of long‐term datasets. NEON and TERN couple standardized field‐collected biological data with remotely sensed environmental data to monitor ecological change and thus operate with a similar approach to the goals of ANTOS. Extensive online data portals, data management, and most notably, resource accessibility, are key to their ongoing success. These global examples highlight that such programs are not only achievable but are capable of revolutionizing how we collect and manage long‐term datasets into the future.

**FIGURE 6:**
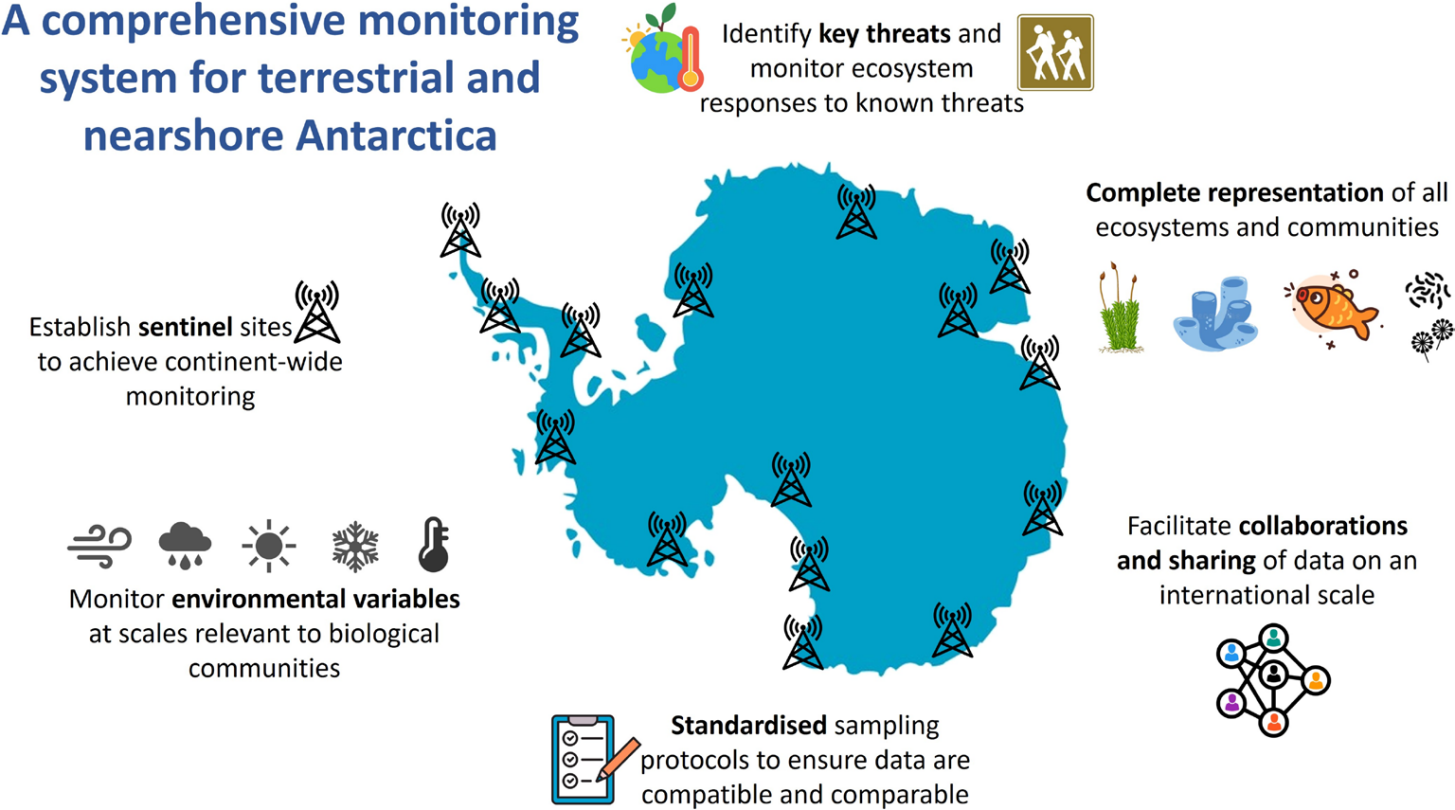
Recommendations for improved outputs from long‐term monitoring of Antarctic nearshore and terrestrial ecosystems based on current limitations and gaps identified in this review.

To improve monitoring outcomes and manage current challenges facing Antarctic biodiversity, we make the following recommendations to address the research biases described above (Figure 6).

### 4.2 Standardized Data Collection

Standardized sampling protocols are pivotal in ensuring data compatibility and study comparisons. One major issue of current biodiversity monitoring across Antarctica is method inconsistency. Variability in sample collections and data measurements limits our ability to compare studies between locations. Given the varying levels of expertise, resources, funding, and time in the field available to different research groups in Antarctica, a tiered approach to designing best practice monitoring methodology (e.g., ANTOS) would increase research group buy‐in and participation from all levels. A tiered approach for monitoring is a set of methodologies organized into levels according to resources and expertise. For example, it could detail what minimum level of monitoring can be achieved with access to very little resources (basic platforms and protocols) and how this can be advanced with higher levels of expertise, funding, and resources (intermediate to advanced). Research groups can then tailor the level of monitoring to their budgets and expertise.

### 4.3 Establish Sentinel Sites

The current biases in monitoring locations across Antarctica mean key biological communities are either under‐monitored or entirely unmonitored. This, coupled with the lack of data consistency, means we are not able to detect changes at the continental scale. Implementing designated monitoring sites or representative locations ensures we can achieve continent‐wide monitoring (Borgmeier et al. 2025).

Connectivity between Antarctic communities, specifically of vegetation, invertebrate, and marine communities, is extremely poor. The environment in which these organisms and communities live means recruitment, dispersion, and migration are limited (Lee, Waterman, et al. 2022). Across the continent, communities are responding to the disparate changes and pressures they face. Baseline surveys and proposed monitoring sites should be initially focused on areas of high ecological significance, such as the ACBRs, MPAs, and ASPAs (Figure 3; Patterson et al. 2024; Terauds and Lee 2016; Walshaw et al. 2024). Although the current ACBR system inevitably has inherent taxonomic bias due to the availability of biological records, it provides an appropriate framework to attempt to improve coverage of LTM across the continent (Figure 6; Borgmeier et al. 2025; Terauds et al. 2025).

### 4.4 Monitor Environmental Variables at Relevant Scales

Many studies did not couple biological data with environmental variables (Tables 1, 2, 3). While most studies with environmental data measured variables locally, many relied on data obtained from the nearest, but often quite distant, weather station. Weather station data collection is patchy across Antarctica, and many environmental variables that are measured elsewhere on the globe are not yet measured adequately. This is particularly true for the microclimate variables, which are so relevant to Antarctic biota (Yin et al. 2023). For example, the first global maps of soil temperature published (Lembrechts et al. 2022) did not cover Antarctica because of the low number of entries for this region in the database in 2021. The current dataset still contains relatively few microclimate records for Antarctica; five are from the peninsula and two from the continent (https://www.soiltempproject.com/the‐soiltemp‐database) (Beugnon et al. *pers. comm*.). Similarly, compared to arctic and alpine locations, permafrost monitoring is limited in Antarctica (Hrbáček et al. 2023; Kubiszewski et al. 2024).

Given how variable regional climate and other environmental data can be (Stenni et al. 2017), it is important researchers have access to locally measured variables. Without such independent variables, it is impossible to understand why ecological communities may be changing, or to predict how they may change into the future. Monitoring environmental variables at local scales, e.g., at the exact monitoring location and not the nearest weather station which could be hundreds of kilometers away, captures the local variability the flora and fauna of the region is responding to. An example of coordinated efforts to address this gap is proposed by ANTOS where climate sensors are installed at sentinel sites in Antarctica to measure and make publicly available biologically relevant environmental data in real‐time. Other coordinated programs already achieving similar goals across the globe, but outside Antarctica, are NEON (Keller et al. 2008) and TERN (Thurgate et al. 2017).

### 4.5 Monitoring Key Biodiversity Drivers

Most publications followed population abundance trends over time. However, given the lack of local environmental data, these trends are difficult to link to some of the key drivers of biodiversity across Antarctica. Less than 10 publications monitored species invasions, or changes to community structure and function in response to contamination, sea ice change, and/or ocean acidification. Already, the establishment of non‐native species has been documented in both terrestrial (Bartlett et al. 2020; Molina‐Montenegro et al. 2014) and marine (Deregibus et al. 2017) communities, in addition to increasing propagule pressure (Huiskes et al. 2014), measures of changes in community diversity and function are important. Future monitoring should place emphasis on identifying key threats to biodiversity loss and monitoring ecosystem responses to known threats.

### 4.6 Data Sharing

Many nations have National Antarctic Data Centers to facilitate their obligation to the Antarctic Treaty, which states that “scientific observations and results from Antarctica shall be exchanged and made freely available” (Article 3). Although there are many examples of data sharing initiatives in Antarctic science, e.g., Southern Ocean Observing System (SOOS) (https://soos.aq/data/soosmap), Palmer and McMurdo LTER programs (https://lternet.edu/site/), Scientific Committee on Antarctic Research Antarctic Biodiversity Portal (https://www.biodiversity.aq/), and some in their early stages, e.g., the ANTOS database for Antarctic‐wide long‐term datasets for nearshore and terrestrial ecosystem monitoring (http://www.antosdb.org/) and the East Antarctic Monitoring Program (via https://data.aad.gov.au/), ecological monitoring data are often not uploaded to public archives. A lack of physical evidence and genetic data can hinder the reliable integration of LTM data into databases. Typically, if trends in data are not published, they remain unknown to the general scientific audience. Although environmental data is becoming more available (Murray et al. 2018), there still remains the issue of incompatibility, owing to different methodologies and because complex data lack metadata explanations and descriptions, and thus are not easily understood by those outside of the research group who collected it. The adoption of data archives encourages international collaboration and reduces repetitive data collection by independent research groups.

Given Antarctica's fragile and rare ecosystems, physical sample collections and disturbance from researchers should be kept to a minimum to comply with the Antarctic Treaty principles, which were designed to protect these communities. Data sharing, in addition to sample sharing across international research groups, will enable sampling to be done minimally, more efficiently, and will optimize collaboration and research expedition outcomes.

## 5 Conclusions

LTM is essential in a time of ecosystem modification, especially under rapid global change. Currently, monitoring of biota across Antarctica is limited, with a stark bias towards charismatic species, such as penguins and mammals. Antarctic monitoring studies are also spatially limited and lack methodological consistency, thus limiting comparisons between them. Developing an Antarctic‐wide observation network, such as ANTOS, will allow harmonized monitoring of Antarctica's flora and fauna via standardized protocols, sentinel site designation, identifying key threats, and coupling biological data with locally measured environmental data. Most importantly, such a system will provide the platform for international collaborations and data sharing to provide a circum‐Antarctic view of environmental change and biological responses.

## Author Contributions

**Shae L. Jones:** conceptualization, data curation, formal analysis, investigation, methodology, visualization, writing – original draft, writing – review and editing. **Diana H. King:** conceptualization, data curation, investigation, methodology, software, validation, visualization, writing – review and editing. **Vonda J. Cummings:** investigation, validation, writing – review and editing. **Sharon A. Robinson:** conceptualization, funding acquisition, investigation, project administration, validation, writing – review and editing. **Melinda J. Waterman:** conceptualization, data curation, formal analysis, funding acquisition, investigation, methodology, project administration, supervision, validation, visualization, writing – original draft, writing – review and editing.

## Conflicts of Interest

V.C. and S.A.R. are executive committee members, and M.J.W. is a committee member of the SCAR ANTOS Expert Group.

## Supporting information


**Table S1.** A combination of the keywords used in the initial literature search conducted in 2020 using SCOPUS.
**Figure S1.** Locations of long‐term biological monitoring sites in published studies in relation to research stations and facilities in Antarctica.

## Data Availability

Data associated with this article are archived with the Australian Antarctic Data Centre (https://data.aad.gov.au/aadc/) at https://doi.org/10.26179/nvjw‐qf32.
