# Supplementary material for: Research Bias in Long‐Term Monitoring of Antarctic Nearshore Marine and Terrestrial Biota
Source: Glob Chang Biol. 2025 Aug 18;31(8):e70392. doi: 10.1111/gcb.70392 (PMC12360029; doi:10.1111/gcb.70392)
Supplement: Supplementary file 1 — Table S1. A combination of the keywords used in the initial literature search conducted in 2020 using SCOPUS. Figure S1. Locations of long‐term biological monitoring sites in published studies in relation to research stations and facilities in Antarctica. [file GCB-31-e70392-s001.pdf]

Supplementary Information

Research bias in long-term monitoring of Antarctic nearshore marine and terrestrial biota

Shae L. Jones[# 1,2], Diana King[# 1,2], Vonda J. Cummings[3], Sharon A. Robinson[1,2], Melinda J. Waterman[*# 1,2]

1. Securing Antarctica's Environmental Future, University of Wollongong, Wollongong, NSW, Australia
2. Environmental Futures, University of Wollongong, Wollongong, NSW, Australia
3. New Zealand Institute for Earth Science, Wellington, New Zealand

*Corresponding author: Melinda Waterman, T +61 2 4239 2379, melindaw@uow.edu.au
[#]Authors who provided equal contributions

S1 Literature Search Methodology

Criteria to include papers

1. Location in Maritime or Continental Antarctica (not sub-Antarctica)
2. Occurred in nearshore marine (<100 m depth) or terrestrial zones
3. Study compared biological parameters in the field
4. Long-term trend/change/response described
5. Measured biological variables at least three time points spanning at least three years
6. Represented unmanipulated, natural variation in the field. For manipulative field studies, papers were only included if there were controls reflecting natural variation/s

Initial search methodology

- Initial literature search conducted in 2020
    - Search engine = SCOPUS
    - Default search settings (searching within title, abstract, keywords, using boolean AND)
    - Keywords used:

Table S1: A combination of the keywords used in the initial literature search conducted in 2020 using SCOPUS.

'Trend' category	Location	Category	Taxa
Monitoring or trend	Antarctic*	Long-term	Bird*
Trend	East* Antarctic*	Glacial retreat	Adelie penguin*
Change	West* Antarctic*	Biodiversity	Seal*
Shift	Antarctic* Peninsula*	Biota	Vegetation
Response	Coastal Antarctic*	Terrestrial	Moss*
		Intertidal	Lichen*
		Marine	Fung*
		Near-shore or nearshore	Etc for all taxa in Tables 1-3

- 2024 update - same as above, but only for papers published after date of initial search

Broadening the search

- Papers meeting the inclusion criteria were searched to find any reference to monitoring papers which may have been missed in the search

- The same keywords were used in National Antarctic Data Centre catalogues to find relevant data or papers. For example, we searched the Australian Antarctic Division (https://data.aad.gov.au/) and British Antarctic Survey (https://data.bas.ac.uk/) metadata databases, and the Palmer and McMurdo Antarctic Long Term Ecological Research (https://lternet.edu/using-lter-data/, https://edirepository.org/, https://search.dataone.org/profile/LTER, https://mcm.lternet.edu/data) program websites to find relevant papers
- The ANTOS, AntERA and AntEco Scientific Committee of Antarctic Research (SCAR) expert group mailing lists were emailed in 2020 to illicit responses from the research community regarding unpublished research and papers in languages other than English
- Conference presentations at SCAR and the Ecological Society of Australia meetings in 2020, SCAR in 2023 and several other small meetings and workshops with ANTOS included requests for knowledge of research or papers

<u>Final search - 2025</u>

- SCOPUS search was revised in 2025
- Search:
    - TITLE-ABS-KEY (long-term OR "long term" OR monitor* OR trend* OR change* OR shift* OR response* OR vari* OR observation*) AND TITLE-ABS-KEY (antarctic* OR "east* antarctic*" OR "west* antarctic*" OR "antarctic* peninsula*" OR "coastal antarctic*") AND TITLE-ABS-KEY (bio* OR ecolog* OR vegetation OR lichen* OR bryophyt* OR moss* OR liverwort* OR alga* OR thallophyt* OR pearlwort* OR colobanthus OR hairgrass* OR deschampsia OR fung* OR bird* OR penguin* OR seal* OR *flora* OR *fauna* OR arthropod* OR *invertebrat* OR nematod* OR "poa annua" OR pygoscelis OR aptenodytes OR eudyptes OR petrel OR shag OR tern OR skua OR gull OR sheathbill OR fulmar OR fish* OR bryozoan* OR *phyto* OR seaweed* OR *plankton*) AND (LIMIT-TO (SUBJAREA , "AGRI") OR LIMIT-TO (SUBJAREA , "ENVI") OR LIMIT-TO (SUBJAREA , "MULT")) AND (LIMIT-TO (PUBSTAGE , "final")) AND (LIMIT-TO (DOCTYPE , "ar") OR LIMIT-TO (DOCTYPE , "dp")) AND (EXCLUDE (EXACTKEYWORD , "Atlantic Ocean") OR EXCLUDE (EXACTKEYWORD , "Arctic") OR EXCLUDE (EXACTKEYWORD , "Indian Ocean") OR EXCLUDE (EXACTKEYWORD , "Australia") OR EXCLUDE (EXACTKEYWORD , "Biogeochemistry") OR EXCLUDE (EXACTKEYWORD , "Stable Isotope"))
    - 11,327 documents were found
    - 11,310 remaining after duplicate removal
    - This search captured 85 papers already included from earlier search stages

Inclusion terms:

- Papers were marked for manual assessment if they included any of the following terms in **abstract, title or keywords** - these papers were assessed manually even if marked for exclusion in the below steps
    - Palmer AND LTER
    - “Latitudinal Gradient Project”
    - “long term” OR long-term OR longterm
    - years
    - seasons
    - summers
    - decad*

- repeat
- “time series”
- monitor* AND (species OR communit* OR population OR ecosystem)
- abundance AND (species OR communit* OR population OR ecosystem)
- distribution AND (species OR communit* OR population OR ecosystem)
- growth AND (species OR communit* OR population OR ecosystem)
- “population change”
- “population response*”
- “population trend”
- consecutive
- “species composition”
- “species distribution”
- “species abundance”
- community AND composition
- community AND change
- temporal
- baseline

Exclusion terms:

- Papers were marked for exclusion where the **publication** contained any of the following:
    - geo*
    - chemi* (to cover chemistry and chemical)
    - agri*
    - *tech*
    - behaviour* OR behavior*
    - aquaculture
    - telemetry
    - astrobiology
    - atmos*
    - CCAMLR
    - chemos*
    - whaling
    - earth (except “Earth’s Future”)
    - anatom*
    - agron*
    - paleo* OR palaeo*
    - toxic*
- Papers were marked for exclusion where the **title** contained any of the following:

- subantarctic* OR sub-Antarctic*
- touris*
- lipid*
- geolog*
- nothofagus
- *america*
- paleo* OR palaeo*
- “deep water”
- “Continental shelf”
- Argentina
- Chile*
- Finland
- Australia*
- New Zealand
- Russia*
- Africa
- *cene
- review
- *zoic
- fossil
- pollut*
- genome
- influenza
- acoustic
- experiment*
- model*
- diesel
- contamin*
- “Southern Ocean”
- CCAMLR

● Papers were marked for exclusion where the **keywords** contained any of the following:

- sub Antarctic* OR sub-Antarctic*
- South Georgia
- Falkland
- South Sandwich Island
- Kerguelen
- Marion
- Prince Edward
- Bouvet
- Crozet
- Heard AND Island
- Macquarie Island
- Campbell
- Snares
- Antipodes
- CCAMLR
- touris*

● Papers were marked for exclusion where the **abstract** contained any of the following:

- “except Antarctica”
- acoustic
- atmospher*
- cruise or voyag*
- fossil
- *zoic
- 14C
- “sea level”
- catalys*
- pollut*
- Patagonia
- India*
- Atlant*
- Namib*
- in vitro
- market*
- clon*
- engineer*
- CO2
- “New Zealand”
- palaeo* OR paleo*
- isolates
- “Treaty System”
- Mediterranean
- carbohydrate*
- Tierra del Fuego
- pesticide
- ATPase
- captiv*
- osteolog*
- “fatty acid”
- crops

- genotyp*
- fluorine
- “Net Primary Productivity”
- fractionation
- patholog*
- “dissolved organic carbon”
- (DOC)
- “deep ocean”
- biogeochem*
- catch AND effort
- CCAMLR
- chemical
- “Continental Shelf”
- expedition
- fishing
- holocene
- isotop*
- “new species”
- Pacific
- taxonom*
- δ
- whal*
- evolution
- reconstruct*
- enzyme*
- Tasmania
- abyssal
- sulfur OR sulphur
- “human health”
- aerosol
- agricultur*
- forest*
- “air sampl*”
- ioniz* OR ionis*
- extraterrestrial OR exobio*
- radioactive
- telemetry
- aquarium
- bioaccumulat*
- polycyclic aromatic hydrocarbon
- martian
- politic*
- astronom*
- circadian
- calls
- chemos*
- mekong
- radiocarbon
- chromosome
- antifreeze
- lipid
- protein
- model*
- manipulat*
- grounding* OR grounded
- metabol*
- “deep water” OR “deep-water”
- energy
- irradiance
- virus OR viral
- “metal level*” OR “metal concentration*”
- contamina*
- adipose
- diurnal

Final results 2025

- The dataset of long-term monitoring studies, locations, study years, biota groups, species, biological parameters and any included environmental variables is publicly available at doi:10.26179/nvjw-qf32 (Waterman et al., 2025).

S2 Distance of LTM sites to research station

Coordinates provided in the included long-term monitoring publications were extracted, mapped using QGIS software v3.42 and compared with locations of research stations and facilities used by National Antarctic Programs for science and science support activities (COMNAP, 2025). Average GPS points were calculated for regions where data analyses were based on pooled data from that region. For example, GPS points were averaged within each of the five regional populations compared by Southwell et al. (2015). GPS points were estimated for LTM studies that did not provide GPS coordinates but mentioned specific locations in text, tables or figures (e.g. point, cove, bay, ASPA etc). Some publications referred to distance to station only in text or figures and were hence included in the final calculation. Publications that did not provide or were not clear on the locations of LTM sites or their distance/s to research station were excluded from this analysis. There were approximately 230 LTM unique sites analysed.

Distance to research station was calculated using the 'buffer' tool on the generated QGIS map. Buffer zones of 5, 10, 20 and 50 km from research stations were analysed for comparison with LTM site locations. The 5 km and 20 km buffer zones align to station footprint zones chosen by Pertierra et al. (2017) and Brooks et al. (2019) who used 5 km and 20 km, respectively. The 'select attribute by location' feature on QGIS was used to layer the LTM coordinate points over the different buffers and count which sites overlapped with the research station zones selected. Sites were counted as being within the research station footprint if they overlapped with the buffer zone. Common LTM sites (e.g. same GPS coordinates) were only counted once.

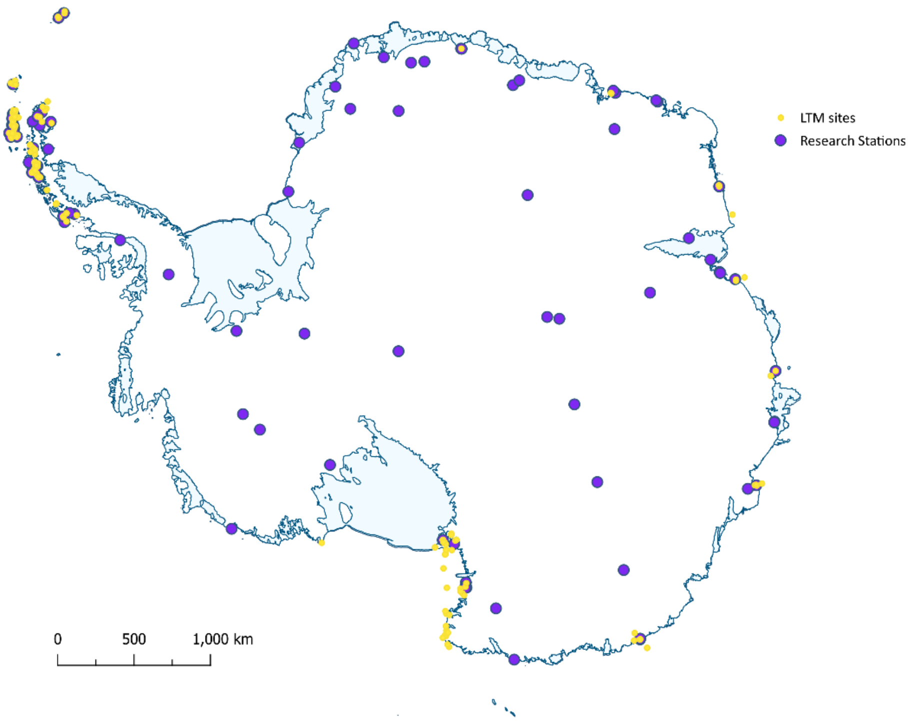

Figure S1: Locations of long-term biological monitoring sites (yellow dots) in published studies in relation to 'open' or 'temporarily closed' research stations and facilities (purple dots) in Antarctica. Geospatial data of Antarctic Facilities were obtained from COMNAP (2025).

References:

Brooks, S. T., Jabour, J., van den Hoff, J., & Bergstrom, D. M. (2019). Our footprint on Antarctica competes with nature for rare ice-free land. *Nature Sustainability, 2*(3), 185-190. doi:10.1038/s41893-019-0237-y

COMNAP. (2025). Council of Managers of National Antarctic Programs (COMNAP) Antarctic Facilities List. Retrieved from: https://www.comnap.aq/s/Facilities_Nov2024.csv

Pertierra, L. R., Aragón, P., Shaw, J. D., Bergstrom, D. M., Terauds, A., & Olalla-Tárraga, M. Á. (2017). Global thermal niche models of two European grasses show high invasion risks in Antarctica. *Global Change Biology, 23*(7), 2863-2873. doi:10.1111/gcb.13596

Southwell, C., Emmerson, L., McKinlay, J., Newbery, K., Takahashi, A., Kato, A., . . . Weimerskirch, H. (2015). Spatially extensive standardized surveys reveal widespread, multi-decadal increase in East Antarctic Adélie penguin populations. *PLoS ONE, 10*(10). doi:10.1371/journal.pone.0139877

Waterman, M., King, D., Jones, S., Cummings, V. and Robinson, S. (2025) Long-term monitoring studies on Antarctic nearshore marine and terrestrial ecosystems, Ver. 1, *Australian Antarctic Data Centre*. doi:10.26179/nvjw-qf32
